# Native Burmese pythons exhibit site fidelity and preference for aquatic habitats in an agricultural mosaic

**DOI:** 10.1038/s41598-021-86640-1

**Published:** 2021-03-29

**Authors:** Samantha Nicole Smith, Max Dolton Jones, Benjamin Michael Marshall, Surachit Waengsothorn, George A. Gale, Colin Thomas Strine

**Affiliations:** 1 School of Biology, Suranaree University of Technology, Nakhon Ratchasima, Thailand; 2 Sakaerat Environmental Research Station, Thailand Institute of Science and Technological Research, Nakhon Ratchasima, Thailand; 3 School of Bioresources and Technology, King Mongkut’s University of Technology Thonburi, Bangkok, Thailand

**Keywords:** Ecology, Behavioural ecology, Ecological modelling, Tropical ecology

## Abstract

Animal movement and resource use are tightly linked. Investigating these links to understand how animals use space and select habitats is especially relevant in areas affected by habitat fragmentation and agricultural conversion. We set out to explore the space use and habitat selection of Burmese pythons (*Python bivittatus*) in a heterogenous, agricultural landscape within the Sakaerat Biosphere Reserve, northeast Thailand. We used VHF telemetry to record the daily locations of seven Burmese pythons and created dynamic Brownian Bridge Movement Models to produce occurrence distributions and model movement extent and temporal patterns. To explore relationships between movement and habitat selection we used integrated step selection functions at both the individual and population level. Burmese pythons had a mean 99% occurrence distribution contour of 98.97 ha (range 9.05–285.56 ha). Furthermore, our results indicated that Burmese pythons had low mean individual motion variance, indicating infrequent moves and long periods at a single location. In general, Burmese pythons restricted movement and selected aquatic habitats but did not avoid potentially dangerous land use types like human settlements. Although our sample is small, we suggest that Burmese pythons are capitalizing on human disturbed landscapes.

## Introduction

Studying and understanding animal movement allows scientists insight into animal behavior^1^, revealing species’ natural and life histories such as migratory or movement patterns, breeding grounds and seasons, and foraging strategies^2–4^. Animal movement links to resource use such as habitat selection as movement is highly impacted by habitat availability^5^. As humans continue to degrade suitable habitats, understanding relationships between animal movement and habitat selection assists in developing conservation-based actions. Southeast Asia in particular is vulnerable to biodiversity loss and suffers from the highest deforestation rate of any region^6^, with land being transformed to accommodate growing populations, subsequent urban sprawl and agricultural conversion^7,8^. Habitat fragmentation and degradation has variable impacts on species: specialist taxa that require specific habitat types are at higher risk of extinction^9^; while habitat generalists may have ecological traits, or behavioral plasticity allowing them to survive (and even thrive) in fragmented landscapes^10,11^. Carpet pythons (*Morelia spilota*), for example capitalize on suburban areas in Australia by sheltering in vegetation thickets or even in the roofs of domestic residences while preying upon human-reliant species like rats (*Rattus spp.)*, pets, and livestock^12^. This resilience to habitat changes and affinity for human-modified landscapes often leads to human-wildlife conflict in the forms of property damage, livestock loss, and physical injury^13,14^.

Despite some species being able to adapt to human-modified areas, reptiles are among some of the most heavily impacted taxa by habitat encroachment and deforestation and are experiencing global scale population declines^15,16^. In addition to habitat loss, Gibbons and colleagues^15^ list introduced invasive species, pollution, and climate change as major factors contributing to population declines. Trade in live reptiles has led to collection from the wild to support the exotic pet trade, and may be the second greatest threat to reptile conservation^17^. Even with widespread population declines, there has been limited research effort dedicated to studying reptile ecology^15,17^ which in turn has resulted in vast knowledge gaps. The paucity of ecological studies on reptiles often means that even seemingly “well-known” species have limited available information about their population dynamics, distributions, resource use and demographics. By studying how reptiles (particularly species prone to conflict such as venomous snakes and large constrictors) move and select areas near humans, we can make recommendations to policy makers and local community stakeholders based on sound science to mitigate conflict events. Furthermore, there is an increasing need to investigate the ecology of reptiles in tropical regions undergoing rapid anthropogenically induced landscape transformations.

The Burmese python (*Python bivittatus* KUHL 1820: 94) is a large (> 5 m), nonvenomous, constricting snake often in conflict with humans. Burmese pythons range throughout Bangladesh, Myanmar, Laos, in Thailand north of the Isthmus of Kra, Cambodia, Vietnam, throughout Southern China and in disjunct populations in Northern and Northeastern India and Southern Nepal^18^. In their native range, Burmese pythons occupy diverse habitat types including tropical lowlands, grasslands, forests, agricultural land, aquatic habitats and even green spaces within cities^18,19^. Burmese pythons may persist in such areas due to their broad ecological niche and ability to take a range of prey types including birds, mammals and other reptiles^20,21^. In addition to being widely distributed throughout Southeast Asia, Burmese pythons are also a highly prolific invasive species throughout Southern Florida, USA, most notably in the Florida Everglades, where they have greatly disrupted native ecosystems^22,23^.

The IUCN lists Burmese pythons as Vulnerable^24^, based on heavy harvesting for traditional medicine, the pet and skin trade, and habitat degradation^24^. Burmese pythons also enter human settlements and consume livestock, resulting in human-wildlife conflict, and thus presents further risks to the species^19,25,26^. These instances of contact with humans may contribute to global population declines from persecution^27,28^. For example, in a study investigating interactions between humans and anacondas in South America using internet videos, 52 (~ 16%) of 330 videos showed humans killing anacondas as a result of these interactions^27^. Conflict between pythons and humans may perpetuate hostile attitudes towards snakes and is ever present in Southern Florida.

Nearly all ecological knowledge pertaining to Burmese pythons is from invasive populations in Southern Florida. Most work has aimed to evaluate their impacts on native ecosystems^22,23,29^. Only two studies have published results on native, free-ranging Burmese python movements –one collected data on a single study animal for only 24 days in Hong Kong^25^, the other had two males and two females tracked for un-specified durations in Bangladesh^19^—neither study presented sufficient data to evaluate seasonality, movement patterns, residence time, or further information necessary to make adequate conflict management recommendations. To address this dearth of knowledge, we set out to (1) quantify space use (occurrence distributions during our study) and adult Burmese python movement patterns in a patchy land-use matrix and (2) determine if Burmese pythons select particular habitat features (distance to aquatic agriculture, forest, roads, settlements, terrestrial agriculture, water).

## Methods

### Study site

**Figure 1:**
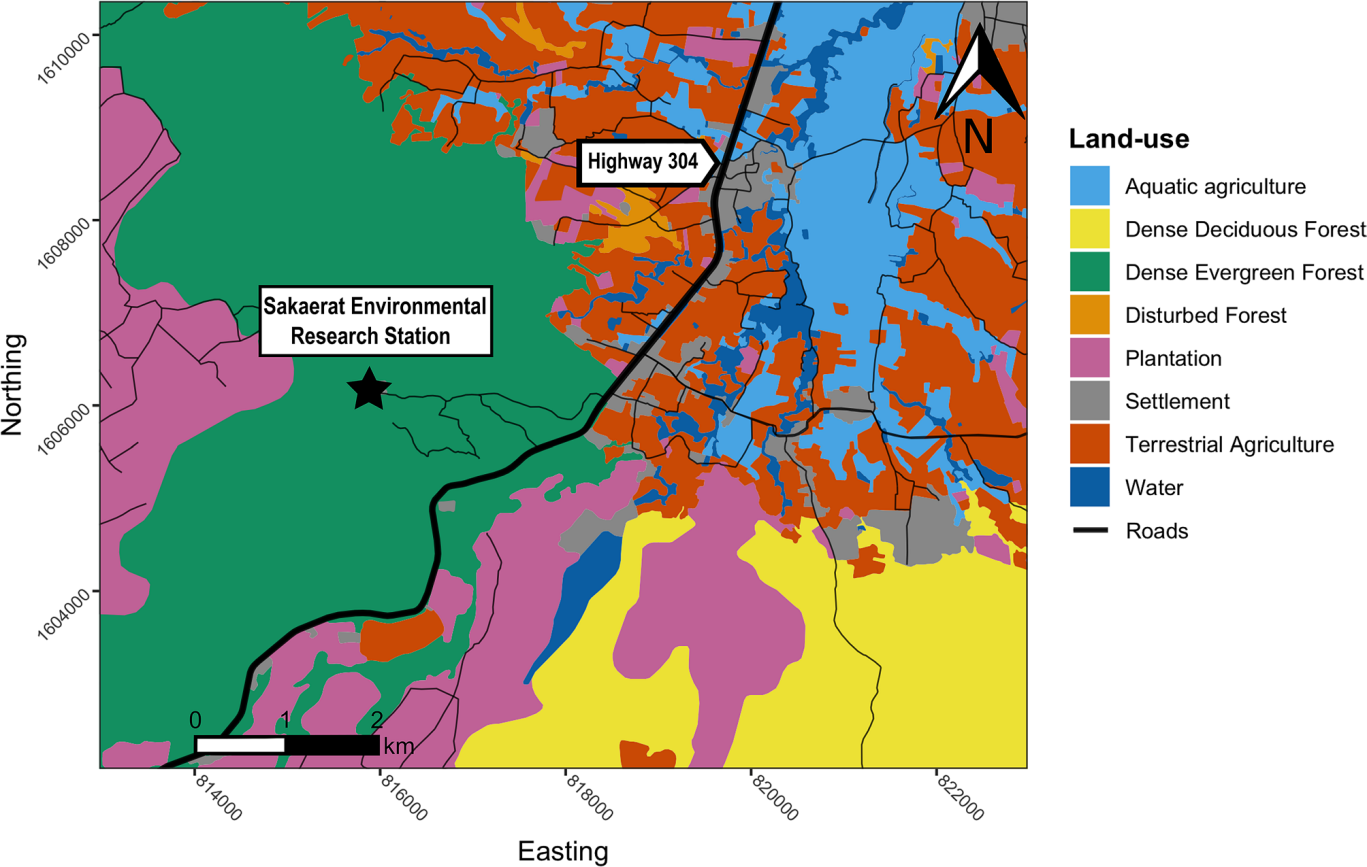
A map illustrating the land-use types spanning the area of which Burmese pythons (*Python bivittatus*) were tracked within the Sakaerat Biosphere Reserve, Nakhon Ratchasima, Thailand. Map created using R v.3.6.3 (https://www.r-project.org/) in RStudio v.1.2.1335 (https://rstudio.com/) in combination with Inkscape v.1.0.2 (https://inkscape.org/).

We monitored all study animals within the Sakaerat Biosphere Reserve in Nakhon Ratchasima Province, Thailand (14.44–14.55° N, 101.88–101.95° E) from 2018-09-29 to 2020-07-22 (Supplementary Fig. 1). The reserve consists of an 8000 ha protected core area with distinct forest types: dry evergreen forest (60%), dry dipterocarp forest (18%), and small patches of reforested area, grasslands, and bamboo forest making up the remaining area (22%). Sakaerat Environmental Research Station rangers regularly patrol the core area to prevent wildlife poaching. Adjacent to the protected area, there is an unprotected buffer zone and transitional zone that together make up 36,000 ha. The buffer zone encompasses stretches of unprotected or otherwise disturbed forests with patches of plantation forest regrowth scattered throughout. Agriculture and human settlements dominate the transitional zone, with several manmade canals and water bodies segmenting the study area (Fig. 1). Most of the agriculture is cassava, followed by rice paddy, sugarcane and corn fields. There are three seasons in our study site defined by daily mean temperature and rainfall; hot (mean: 33.8 ± standard error [SE] 2.8 °C; 2.5 ± 7.9 mm rainfall; 16 Mar. to 30 Sept.), wet (29.9 ± 2.2 °C; 5.9 ± 11.1 mm rainfall; 1 Oct. to 31 Dec.) and dry (29.0 ± 3.5 °C; 0.2 ± 0.8 mm rainfall; 1 Jan. to 15 Mar.;^30^).

### Study animal capture, processing and implantation

We captured all study animals opportunistically, through visual encounter surveys, or via notification from residents in the surrounding villages (Supplementary Table 1). Once we captured an individual, we collected biometric data at our field laboratory at the Sakaerat Environmental Research Station. We gave all snakes a unique ID using the first two letters of the genus and specific epithet and a number based on capture sequence (PYBI###). To accurately measure animals with minimal stress, we used isoflurane, an inhalant anesthetic. Once the snake was fully anesthetized, we collected biometric measurements including snout-to-vent length (SVL; mm), tail length (TL; mm) and mass (g) (Supplementary Table 2). We determined the sex of each snake via cloacal probing. We attempted to only implant transmitters into adult females (total length > 2500 m^31^) captured within 15 km of our study area in order to maximize our sample size and strengthen our statistical inference. A licensed veterinarian surgically implanted very high frequency (VHF) transmitters (AI-2 T or SI-2 T, Holohil, Carp, Ontario, Canada) following Reinert and Cundall^32^ using isoflurane anesthesia. Transmitters did not exceed 0.26% of the snake’s mass. Surgeries were not always conducted immediately after capture because of limited veterinarian availability. Following surgery, we monitored and released snakes within 24 h of implantation to provide maximum opportunity for natural thermoregulation and to facilitate healing. We released snakes at their initial capture site; however, in instances where snakes were captured from local residences (n = 4), we were unable to use the direct capture location. In these cases, we released snakes in areas that were as close as possible to the initial capture site with ample vegetation and cover. We released snakes an average of 149.12 m ± SE 49.49 m (range 0–393 m) from their initial capture site (Supplementary Table 1). We began collecting data on implanted snakes the day following their release; as we observed no obvious subsequent behavioral changes between initially implanted and acclimated snakes, we therefore included all tracking data in our analyses.

### Radio-tracking

We radio-tracked study animals during daylight hours (06:00–18:30) approximately once daily. However, there were occasional instances that we were unable to track study animals due to staff limitations, equipment malfunction, difficulty obtaining telemetry signals, inclement weather (which damages receiving equipment) or because an animal was in lab for transmitter reimplantation. On average, snakes had 17 ± 7 missing data points throughout the study. Despite these instances, we maintained a mean tracking time lag (time between telemetry fixes) of 25.43 ± 0.32 h for the entire study duration (Supplementary Fig. 2). In order to increase location accuracy, we homed-in on tracked snakes to pinpoint their exact location. In the event that we could not directly approach the snake (e.g., snake was in a water body, terrain did not allow for us to approach) we used three-point triangulations from as close as possible to estimate the snake’s location. Upon locating the snake, we recorded observation time, Universal Transverse Mercator (UTM WGS-84) points and GPS accuracy (m) with a hand-held GPS units (Garmin 64 s; Garmin International Inc., Olathe, Kansas, USA). To decrease disturbance, we retreated at least five meters after locating a snake to record datapoints.

### Space use/occurrence distributions analysis

We used R v.3.6.3^33^ using RStudio v.1.2.1335^34^ for all analyses and visualization. A comprehensive package list is available in the supplementary material. We quantified space use via occurrence distributions (also referred to as confidence areas) with dynamic Brownian Bridge Movement Models (dBBMMs). We elected to use dBBMMs to quantify space use over more traditional (often termed home range) estimation methods such as minimum convex polygons (MCPs) and kernel density estimates (KDEs, which produce utilization distributions) due to the fact that these traditional methods tend to greatly overestimate or underestimate space use^35,36^. In contrast, dBBMMs create a more refined visualization of animal movement by taking into account the order of animal relocations and the duration of time spent at each location^37^. The dBBMMs require window size and margin size input to estimate an animal’s movement capacity (motion variance) and to detect changes in movement based on behavioral states. The window size, and the datapoints held within, calculate the animal’s movement capacity on a rolling basis. The margin size operates within the window size and is used to detect changes in movement states (e.g., different behaviors detectable in movement patterns) allowing for changes in motion variance within a given window. The window and margin size used in dBBMMs should be biologically relevant to the study species and sampling regime (and should match with the temporal frequency of behavioral changes)^37^. To set our window and margin size we followed methodology suggested by Marshall et al.^30^ and selected a window and margin size based off of the typical movements of Burmese pythons during the tracking duration. Given that tracked Burmese pythons would often remain stationary for long periods of time (~ two weeks), we selected a window size of 15 telemetry fixes to detect longer term movement behavioral shifts. For the margin size, we selected a margin of three telemetry fixes (both window and margin size must be odd numbers) as we were able to detect behavioral changes (stationary vs. active) over the course of approximately two telemetry fixes.

The dBBMMs also provide motion variance estimates over the course of the tracking duration. Motion variance is essentially a measurement in the changes of movement intensity during the tracking period. Visualizing motion variance can also be helpful to identify spikes or changes in movement and therefore can provide insight into changes in behavioral states. We explored seasonal patterns in motion variance for three of our snakes tracked a full year or more and through all three seasons (PYBI021, PYBI022 and PYBI029). Marshall et al.^30^. determined date ranges and environmental thresholds via cluster analysis for the three seasons occurring in our study site which we used to determine our seasonal delineations. We produced telemetered Burmese python occurrence distributions and motion variance with dBBMMs with the *move* package v.4.0.2^38^. To extract contours (90, 95, 99%) or estimates of space use from the occurrence distributions and calculate areas, we used *adehabitatHR* v.0.4.18^39^ and *rgeos* v.0.5.3^40^ packages.

We also investigated individual site fidelity using recursive analysis in package *recurse* v.1.1.2^41^. In ecology, recursive analysis is used to explore recursive movement patterns, or tendencies for animals to return to certain areas (i.e., shelter sites, breeding grounds, foraging areas)^42^. For each individual, we took the mean GPS error to determine the radius for sites to be used in the analysis. Revisits occurred when an individual moved away from a location and then returned at any point after 24 h had elapsed.

### Integrated step selection analysis

To investigate Burmese python habitat selection, we used integrated step selection functions (ISSFs) on both an individual and population level (focusing only on the sample of pythons using agricultural systems, which removed one individual that consistently used forest habitat). Integrated step selection functions are a type of resource selection function that use observed locations or steps from tracking data. Using the observed steps, additional or available steps are randomly generated from a distribution of these observed steps, taking distance between steps and direction of steps into account^43^. In integrated step selection functions, predictor covariates, in this case Euclidean distance to certain land use types, are compared against each other to identify which best predicts observed locations (also termed used locations) versus the available locations (locations that were not observed to be used during study). Integrated step-selection functions are especially useful when working with movement data because of how they compare used versus available habitat features on a step-by-step basis, albeit with the assumption that temporal resolution of tracking/data approximates the study animals’ decisions regarding habitat use.

For individual selection, we created integrated step selection functions using package *amt* v.0.0.6^44^. We used Euclidean distance to habitat features (water bodies, forest, terrestrial agriculture, human settlements, roads and aquatic agriculture) as covariates in our ISSFs. To create ISSFs, we modified code from Marshall et al.^45^. We used a land use shapefile from the Thai Land Development Department (2017) separated into several land use categories to create raster layers with a cell size of approximately 10 m. We converted the raster layers from binary raster layers to layers with continuous values by calculating the Euclidean distances to habitat features of interest (i.e., Euclidean distance from individual raster cells or pixels to: water bodies, forests, terrestrial agriculture, human settlements, roads, and aquatic agriculture). We grouped water bodies (ponds, reservoirs, and irrigation canals) with semi-natural areas, which typically consist of overgrown vegetation bordering water bodies as the two habitat features were highly correlated with each other. To avoid zero-inflation of distances to feature values and produce more intuitive effect directions in the models, we inverted all our raster layers. Global positioning system (GPS) trackers can provide high temporal resolution movement datasets; but high temporal resolution data and the high computational costs of ISSF can limit the number of random steps^46^. However, our VHF tracking methods provides a temporally coarse dataset (25 h on average), allowing us to generate more random steps enabling a broad sampling of the landscape and ensuring rare habitats were not missed. We therefore opted to generate 200 random steps for each observed step (i.e., relocation) which optimized sampling while also accounting for the computational power needed for ISSF.

We created ten models including step length and turning angle as covariates. Step lengths were drawn from a gamma distribution and turn angles from a von Mises distribution, both of which were parameterized using steps observed during radio-telemetry tracking. One of the ten models, our null model, only incorporated step length and turning angle to predict movement. We created six models that used a single habitat feature to predict selection, and three models that were multi-factor models which included a combination of the three different habitat features to predict habitat selection (Table 2). To determine model performance, we used Akaike’s Information Criterion (AIC) and we considered models with ∆ AIC < 2 as top performing models.

We expanded our analysis to investigate habitat selection at the population level, including all of our telemetered snakes (with the exception of PYBI060, an exclusively forest animal). We adapted code provided by Muff et al.^47^ who used a mixed conditional Poisson regression model with stratum specific effects, equivalent to an integrated step selection model at the population level with individuals modeled as a random effect. In the Poisson model, both step (strata) and individual are modeled as Gaussian processes. We used the same process to generate 200 random steps as used for the individual level ISSF (Gamma distribution for step length and Von Mises distribution for turn angle). We created six single factor models using the same habitat features and rasters used to explore individual habitat selection (i.e., inverted distance to forest, settlements, roads, water bodies, aquatic agriculture, and terrestrial agriculture), with individual random intercepts and slopes. We used a fixed prior precision of 0.0001 for the stratum-specific random effect (i.e., step), as used by Muff et al.^47^. For the other random slopes (i.e., individual) we used a Penalized Complexity prior, PC(1, 0.05); for fixed effects we used uninformative normal priors Normal(0, 10^3^). These models provided us with the amount of individual variation that exists between the selection of our different habitat types as well as population level estimates of habitat selection. We fitted these Bayesian models via integrated nested Laplace approximations using the *INLA* package v.20.03.17^48^. With all analyses, we have provided all R code with exact model specification at https://doi.org/10.5281/zenodo.4026928.

### Approval for animal use

Our research was permitted by National Research Council of Thailand (NRCT) and by the National Park, Wildlife and Plant Conservation Department, Thailand (DNP) (0002/6115). Our methodology is in accordance with the Ethical Principles and Guidelines for the Use of Animals for Scientific Purposes provided by the National Research Council of Thailand. All work was conducted under Institute of Animals for Scientific Purpose Development (IAD) licensing belonging to C.T.S.

## Results

We tracked seven adult Burmese pythons (six females, one male) over the course of our study period (2018-09-29 to 2020-07-22) (Supplementary Table 2). We set out to track only females; however, due to a misidentification of one individual’s sex (human error during processing), we tracked one adult male. We tracked individuals for a mean of 327 ± SE 85 days (range 41–662 days). On average, Burmese pythons moved 41.56 ± 7.43 m per day (Supplementary Table 3). During this time, we located snakes on average 310 ± 80 times (range 4–631 times) with a mean time lag, or time between data points, of 25.43 ± 0.32 h (range 8.55–452.77 h). Burmese pythons had a mean of 112 ± 33 unique locations (range 23–234 unique locations) and were stationary for approximately 5 days (125 ± 11.6 h; range 24.1–2010 h) at a time.

### Occurrence distribution and motion variance

**Figure 2:**
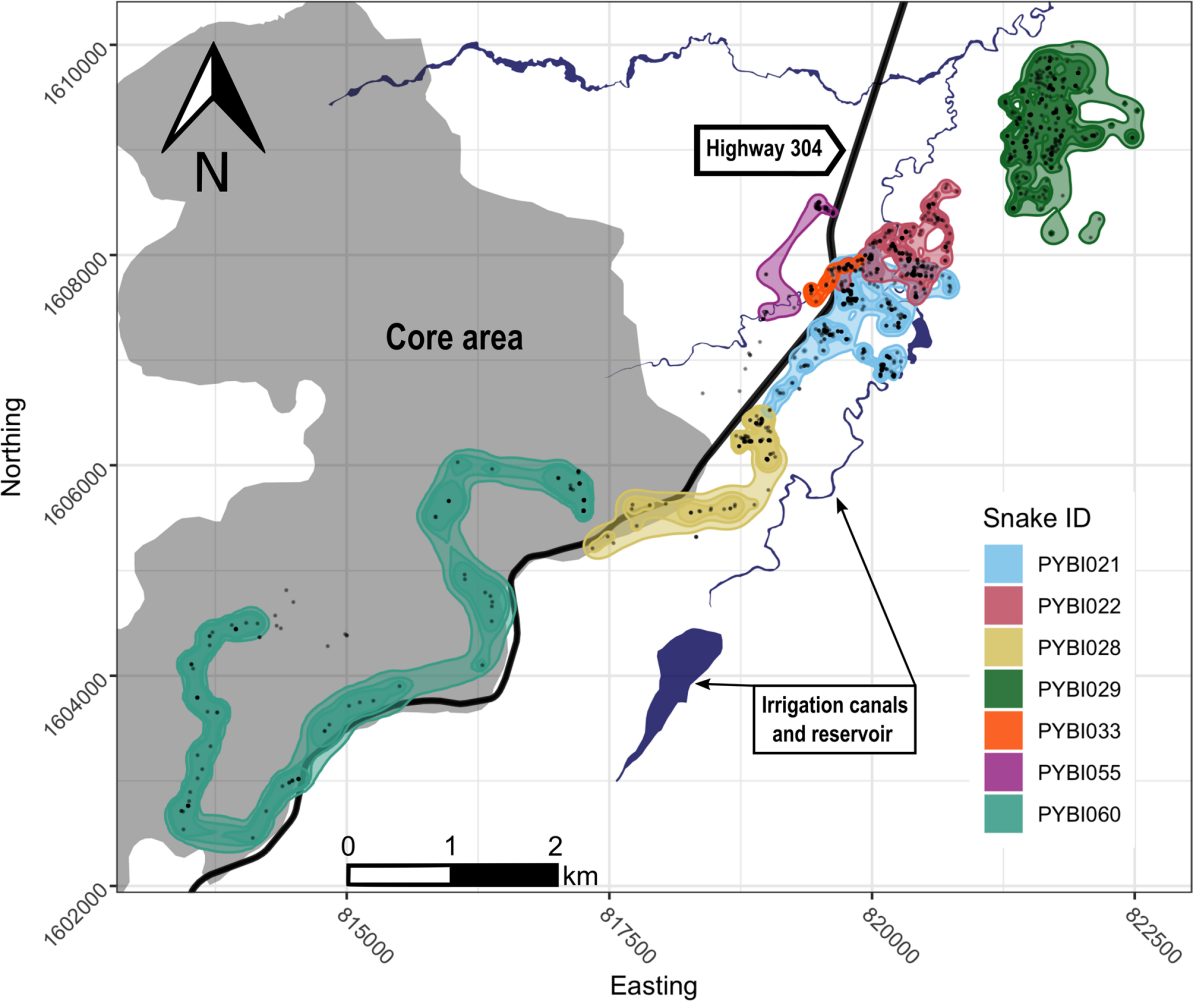
Dynamic Brownian Bridge Movement Models occurrence distributions (95% and 99% confidence areas) for radio-tracked Burmese pythons *(Python bivittatus*) in the Sakaerat Biosphere Reserve, Nakhon Ratchasima, Thailand. Black points on the map denote snake locations from telemetry. Map created using R v.3.6.3 (https://www.r-project.org/) in RStudio v.1.2.1335 (https://rstudio.com/) in combination with Inkscape v.1.0.2 (https://inkscape.org/).

We calculated the occurrence distribution (i.e., estimate of uncertainty surrounding movement paths) for all tracked snakes (Table 1; Fig. 2). On average, the Burmese pythons’ 99% confidence areas were 98.97 ± 35.42 ha. Excluding our single tracked male, female Burmese pythons had a mean 99% confidence area of 100.74 ± 41.86 ha. The largest 99% confidence area (285.56 ha) belonged to PYBI060 who exclusively used the protected biosphere reserve core area. The snake with the smallest 99% confidence area, PYBI033 (9.05 ha), also had the shortest tracking duration (41 days) due to an early transmitter failure. Our tracked male with over six months of data, had one of the smallest 99% confidence areas (88.38 ha). Our longest tracked snake, PYBI021 had a 99% confidence area of 99.94 ha. Movements within the 99% confidence areas showed considerable site fidelity. Five of seven snakes returned to previously used shelter sites (range 4–35 revisits); revisiting sites once every 43.47 ± 14.64 days on average.

**Table 1:** Tracking summary for tracked Burmese pythons *(Python bivittatus*) in the Sakaerat Biosphere Reserve, Nakhon Ratchasima, Thailand.

ID	Sex	Fixes	Start	End	Days	Lag (h)	Relocations	90%	95%	99%	Mean motion variance (m)
PYBI021	F	631	9/29/18	7/22/20	662	25.22 ± 0.33	216	25.27	46.81	94.94	1.74 ± 0.12
PYBI022	F	438	10/25/18	2/23/20	486	26.69 ± 1.11	151	17.7	28.83	53.64	1.24 ± 0.1
PYBI028	M	176	1/9/19	7/16/19	188	25.76 ± 0.79	70	23.66	44.87	88.38	6.27 ± 1.19
PYBI029	F	486	2/23/19	7/22/20	515	25.48 ± 0.83	234	59.16	82.01	139.5	3.67 ± 0.32
PYBI033	F	41	5/18/19	6/28/19	41	24.65 ± 0.61	23	4.81	6.27	9.05	1.09 ± 0.17
PYBI055	F	205	11/12/19	6/6/20	207	24.37 ± 0.33	27	0.08	0.76	21.71	0.63 ± 0.15
PYBI060	F	191	1/5/20	7/14/20	191	24.12 ± 0.25	64	116.9	176.9	285.6	6.06 ± 0.69

Summary includes: sex, F, Female, M, Male; fixes, the number of times a python was located and data recorded; start and end date, month/day/year; mean time lag, time in between consecutive telemetry fixes in hours with standard error; relocations, number of times tracked pythons moved from one location to another; 90%, 95% and 99% occurrence distributions (ha), and mean motion variance with standard error.

**Figure 3:**
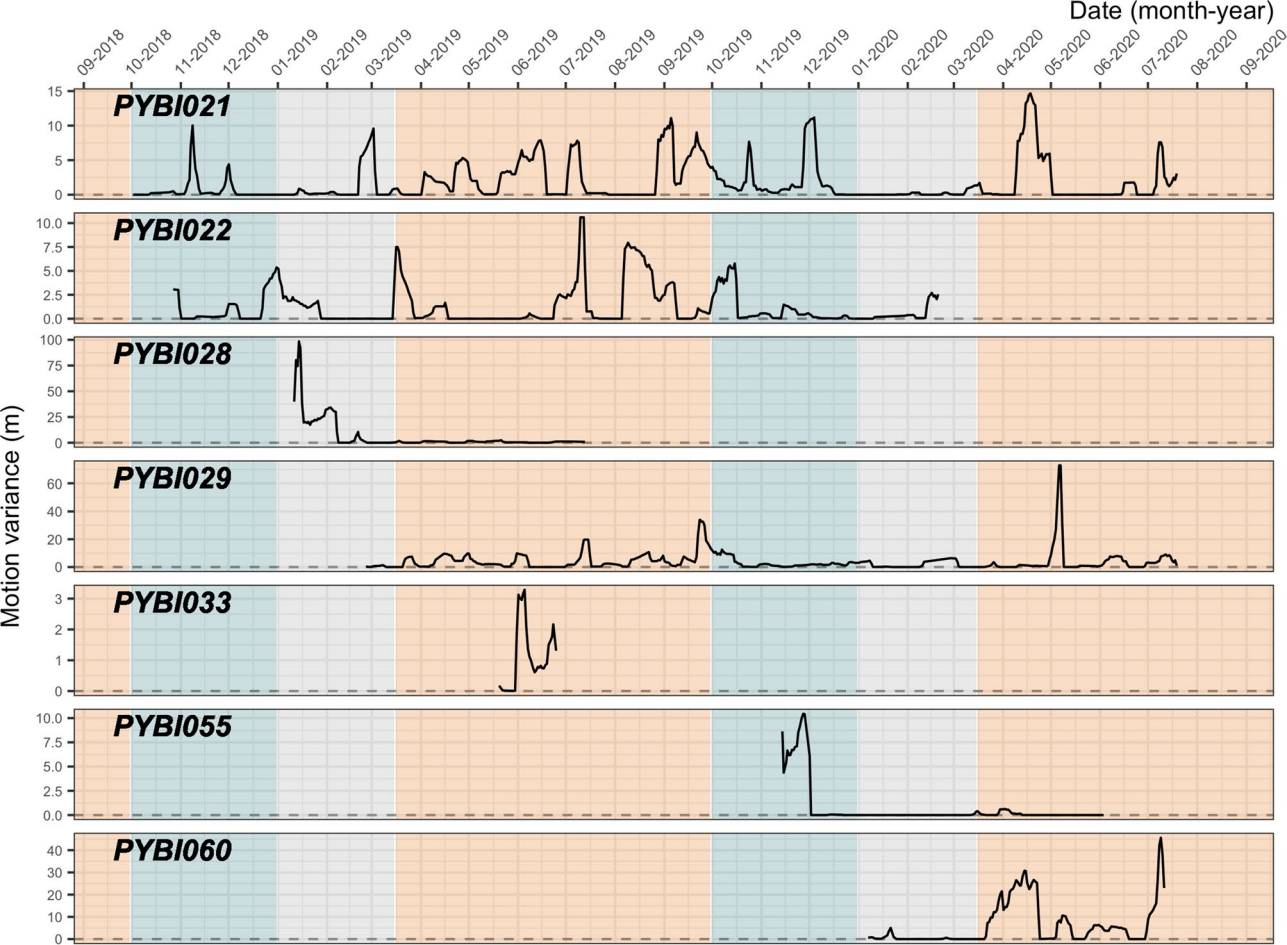
Motion variance for radio-tracked Burmese pythons (*Python bivittatus*) in the Sakaerat Biosphere Reserve, Nakhon Ratchasima, Thailand. Motion variance is plotted for each individual, including one male (PYBI028) across individual tracking duration and season. Blue = wet season, grey = dry season, orange = hot season.

On average, motion variance was low (mean 2.66 ± SE 0.14 m; range 5.53^–05^–98.45 m) (Fig. 3). The individuals with highest mean motion variance were the male, PYBI028 (6.27 ± 1.19 m) and forest dwelling female, PYBI060 (6.06 ± 0.69 m). The lowest mean motion variance was the largest tracked female, PYBI055 (0.63 ± 0.15 m), who remained stationary (range1–84 days spent in a single location) throughout much of her tracking duration during breeding and nesting.

We explored seasonal motion variance differences in three individuals (PYBI021, PYBI022, and PYBI029). Slight visual differences in motion variance are apparent. Motion variance was highest during the hot season (3.12 ± 0.21 m). Mean motion variance was slightly lower during the wet season (1.57 ± 0.12 m) and lowest during the dry season (0.76 ± 0.09 m). We saw that the motion variance for our tracked male, PYBI028, peaked in the dry season before he appeared to greatly reduce his movement immediately prior to the hot season. In contrast, all tracked females tended to limit their movements, showing very few motion variance spikes, during the dry season.

### Integrated step selection analysis

**Figure 4:**
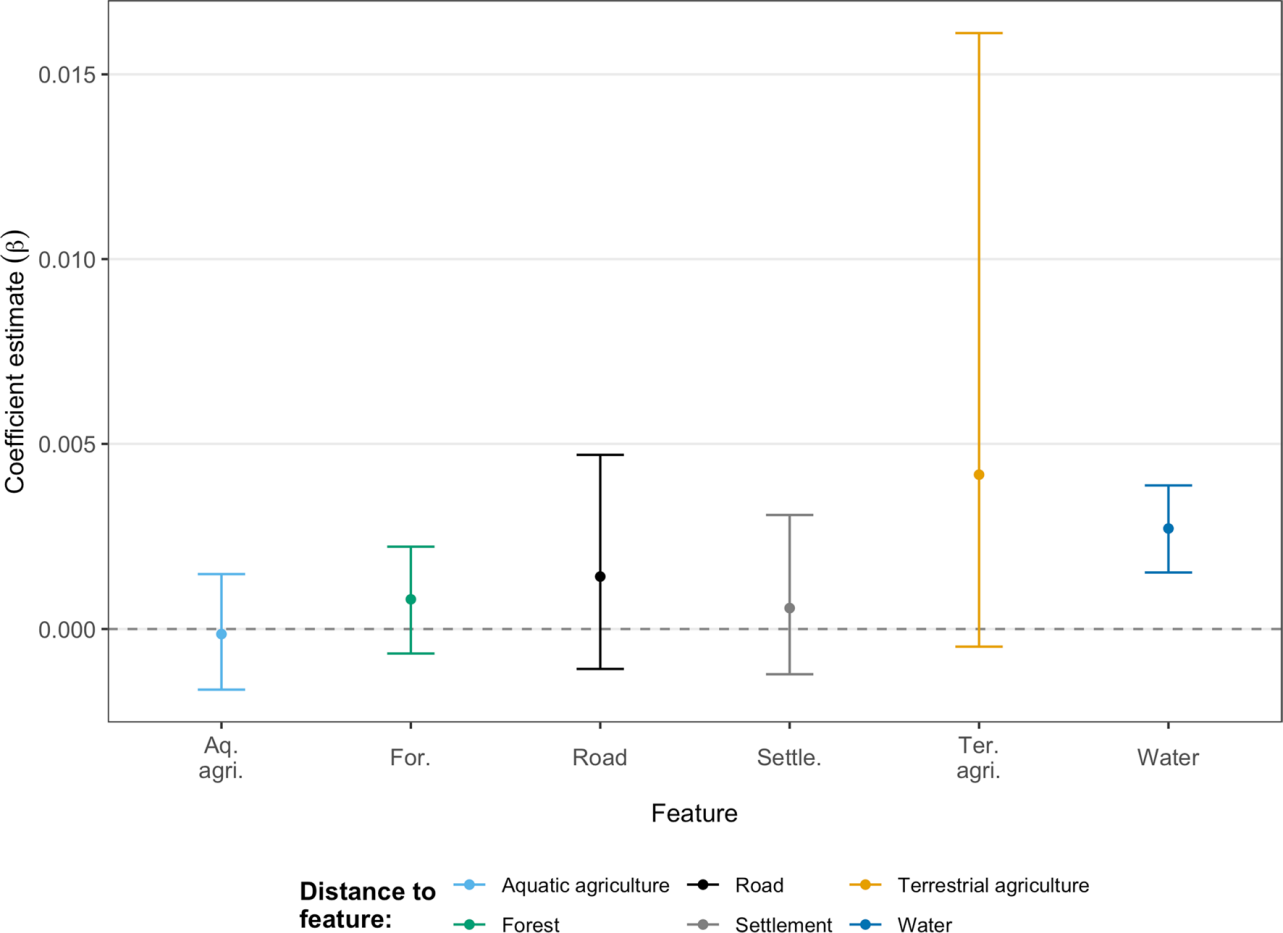
Habitat selection at the population level for all Burmese pythons (*Python bivittatus*) tracked in the agricultural matrix within the Sakaerat Biosphere Reserve, Nakhon Ratchasima, Thailand. Selection is based on distance to habitat features with positive estimates indicating positive association. Error bars indicate 95% credible intervals.

All snakes included in the step selection analysis were associated with aquatic habitats. The population models showed a positive association with water bodies (95% CrI [Credible interval] 0.001–0.004) (Fig. 4; Supplementary Table 4). We failed to detect any unambiguous (i.e., all CrIs overlapped 0) avoidance of human settlements or roads. At the population level, interactions between step length and distance to habitat feature were consistently low (Supplementary Fig. 3).

**Table 2:** Model formulas and $\Delta$ AIC scores for individual integrated step selection functions.

Model	Model formula	PYBI021	PYBI022	PYBI028	PYBI029	PYBI033	PYBI055
1	log_sl*cos_ta + strata(step_id_) (null model)	21.92	8.13	11.78	18.04	2.39	17.05
2	forest + forest:sl + forest:ta	19.12	12.64	7.59	14.27	6.34	21.56
3	settle + settle:sl + settle:ta	17.19	13.74	7.04	20.93	**1.85**	17.43
4	road + road:sl + road:ta	18.46	7.98	12.61	18.18	**1.61**	15.29
5	water + water:sl + water:ta	4.7	**0**	5.32	**0.14**	2.51	5.67
6	aq.ag + aq.ag:sl + aq.ag:ta	19.75	9.83	**0**	22.43	**0.94**	16.46
7	terr.ag + terr.ag:sl + terr.ag:ta	21.46	8.97	11.32	15.72	4.58	20.08
8	road + forest + settle	24.43	11.04	14.83	6.9	6.41	19.24
9	road + terr.ag + water	3.51	6.83	3.62	**1.81**	**1.22**	**0**
10	water + settle + aq.ag	**0**	5.68	10.1	**0**	**0**	6.39

Integrated step selection functions were created using observed steps of radio tracked Burmese pythons (*Python bivittatus*) within the agricultural matrix (i.e., all individuals excluding PYBI060) in the Sakaerat Biosphere Reserve, Nakhon Ratchasima, Thailand. Each model includes interactive effects of turn angle and step length. sl: step length, ta: turn angle, settle: human settlement, aq.ag: aquatic agriculture, terr.ag: terrestrial agriculture. Emboldened text indicates score within < 2 Δ AIC of the model with the most support for a given individual.

**Figure 5:**
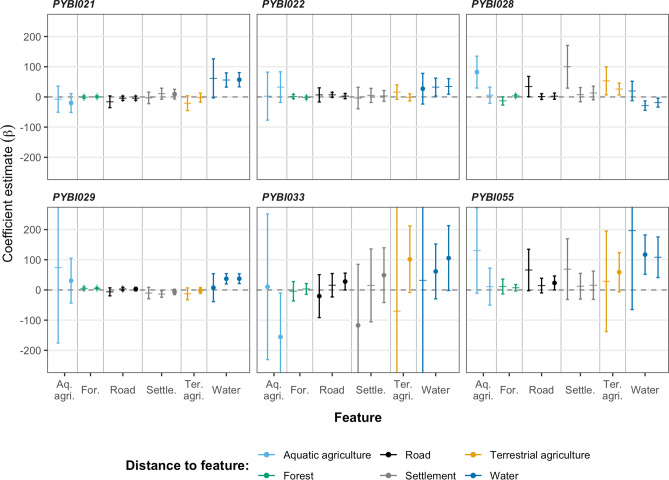
All coefficients from individual integrated step selection functions for Burmese pythons (*Python bivittatus*) tracked in the agricultural matrix (i.e., all individuals excluding PYBI060) within the Sakaerat Biosphere Reserve, Nakhon Ratchasima. Positive estimates suggest association with habitat feature. Error bars indicate 95% confidence intervals. Circles mark the habitat features that were included in models with the lowest AIC score.

Four different models best illustrated habitat selection across individuals (Table 2; Fig. 5), Model five (associated with water), Model six (aquatic agriculture), Model nine (road, terrestrial agriculture and water) and Model 10 (water, settlements and aquatic agriculture). Of these models, Model 10 was the top model for three individuals (PYBI021, PYBI029 and PYBI033) and showed a positive association for the features included in the model (water, settlements and aquatic agriculture). Model six was the top model for our male, PYBI028 and showed aquatic agriculture primarily predicting movements. Model five was a single factor model showing selection for distance to water bodies and was the top model for PYBI022. Model nine was another multi-factor model that incorporated roads, terrestrial agriculture and water to predict selection and was the top model for PYBI055. For several models, confidence intervals for particular individuals were very broad and in some cases overlapped zero, therefore limiting inferences. Model uncertainty likely links to the coarse nature of the dataset and infrequent movement with long stopover times, which results in fewer steps to add into the model. We did not detect consistent interaction between step length and distance to landscape features as there was pronounced individual heterogeneity (Supplementary Fig. 4; Supplementary Table 6). Only our tracked male, PYBI028 showed any interaction between step length and distance to habitat features. Overall, characteristics of steps (length and turning angle) were not affected by land use type; however, location of observed steps were.

## Discussion

Our study is one of the first to investigate the space use, movements, and habitat use of native Burmese pythons. We incorporated innovative methodology (i.e., use of dBBMMs and ISSFs) to explore movement and habitat preference at both the individual and population level, making this study the first ever to use population level ISSF to document the scale of habitat generalism within agricultural land use types in a large, constricting snake. Our results include the movements of several individuals that were concurrently tracked for extended durations (≥ 1 year) revealing seasonal movements. Despite our small sample size, we observed several trends that remained fairly consistent across our study animals. Burmese pythons reused shelter sites and their motion variances suggest that movements were somewhat infrequent, often spending several days in a single location. Within our study site, all individuals (aside from one individual that never left the forest) showed movements associated with habitat features found within the agricultural matrix of the Biosphere reserve’s transitional zone. Of these habitat features, aquatic features (water-based agriculture such as rice paddies and water bodies) appeared to be selected by all individuals to varying degrees.

Previous Burmese python studies (both in their native range and invasive range) used more “traditional” space use estimators (usually referred to as home ranges), such as MCPs and KDEs, which are limited in their ability to be compared across studies because of sensitivity to tracking regime^35,36^. Rather, we measured the occurrence distributions of our tracked snakes during our tracking period. Despite this, we are able to draw some broad inferences between our results and those from studies in their invasive range. For instance, in their introduced range (Florida, USA), Hart and colleagues^49^ found that Burmese pythons used very large areas (mean 2250 ha) and did not exhibit site fidelity, instead individuals made large linear movements (range 110–2430 m), although the methods used to measure space use (MCPs) tend to overestimate area use.

In contrast, our native telemetered pythons moved within much smaller areas. We suspect that there are various reasons as to why movements exhibited by invasive pythons vary greatly from those in Southern Florida. For example, as previously mentioned, traditional space use estimators typically exaggerate areas used, whereas dBBMM’s produce a more refined and conservative estimate^36^. However, due to the stark contrast in our space use estimates (mean: 98.97 ha, 99% contour) and those described by Hart et al.^49^ (mean 2250 ha) it is likely that there are other factors at play. Variable space use between our study and the invasive Florida pythons may be a result of pressures present in our study site that may not be as prominent, or exist, in their invasive range. Our study site is mostly agricultural and human modification can create movement barriers thus impacting both fidelity and movement trajectory^30,50^. For example, Burmese pythons may be altering their movements by spending extended periods sheltering or by making short, infrequent moves to avoid conflict with humans or other anthropogenic risks^10^. Furthermore, we have observed King Cobras preying upon Burmese pythons within the agricultural landscape of our study site. It is possible Burmese pythons remain in human dominated areas to avoid predation from ophiophagus predators^51^. Burmese pythons could be limiting movement to reduce likelihood of encountering predators, thus partially explaining the low motion variance along with their sedentary life history strategy.

Burmese pythons may also alter or restrict their movement to capitalize on prey availability. Human dominated landscapes host potential prey items such as livestock or commensal animals that dietary generalists are able to exploit. Animals often increase or decrease space use in response to resource availability with decreased movement and space use being associated with higher resource availability^52^. Additionally, pythons frequently reused shelter sites, suggesting that certain locations within their observed occurrence distribution may serve as valuable resources in the form of shelter or ambush sites. Surprisingly, the forest-dwelling individual from within the protected area of the biosphere reserve never reused a shelter site, perhaps due to lack of movement barriers or higher availability of ambush and shelter sites. Frequent site reuse by agriculture dwelling individuals suggests that these snakes may reuse refugia in areas with high human activity due to fewer suitable or safe places to shelter in a modified environment. In areas or times of year with few sufficient shelter sites animals may repeatedly return to suitable sites to take refuge^53^. The only other individual tracked within the forest was our male (PYBI028) who briefly moved into a patch of forest during what we assume to be the breeding season (December-March) for Burmese pythons at our study site.

The snakes consistently exhibited low mean motion variances with intermittent activity peaks followed by extended periods of the snake remaining stationary. Burmese pythons are ambush predators^54^, so activity peaks could be a result of searches for new ambush sites and then extended time periods of stationary waiting, capturing and digesting prey (although we never documented successful natural predation incidents). An ambush foraging strategy may not only affect movement patterns and motion variance, but also overall space used by Burmese pythons. For example King Cobras (active foragers), exhibit remarkably higher motion variance (mean 27.9) and utilize larger areas (mean 750.30 ha, 99% contour) within the same study site^30^.

For reproductive females, reproductive behaviors (brooding) further explained long inactive periods with negligible motion variance. Burmese pythons invest substantial maternal care by remaining coiled around their eggs following oviposition until hatching^55^. Two individuals (PYBI022 and PYBI055), nested between April and June and greatly limited their movement during brooding. One individual PYBI055 was within a single burrow for 84 days from late December to mid-March prior to moving to her oviposition site. Several conspecifics entered and left the burrow throughout the 84 days. We suspect that these individuals were likely males following pheromones to the receptive female. Burmese pythons form mating aggregations in their invasive range^56^; and this may have occurred within the burrow; however, there have not been any recorded observations of native Burmese Pythons forming breeding aggregations. When we examine the movements of PYBI022 (another reproductive female) during the same timeframe, we see that there were several locations she visited where she would remain for an extended period of time (14–21 days). Additionally, although infrequent, we did observe molted skin outside of snake shelter sites, suggesting that ecdysis may additionally explain prolonged stationary periods.

Burmese python movements were positively associated with aquatic habitat features such as water bodies (i.e., ponds, irrigation canals) and aquatic agriculture (i.e., rice paddy), suggesting that aquatic elements are important in habitat selection. In their invasive range, Burmese pythons have capitalized on aquatic environments and are thought to be semi-aquatic^57^, which is consistent with our findings of Burmese pythons selecting for aquatic features. We suspect that the Burmese python’s use of connected systems of aquatic agriculture in their native range (this study site, rice paddies and irrigation canals, Thailand) may help explain their success in their invasive range (natural Everglade wetlands, Florida, USA) that is similarly characterized by interconnected wetlands.

In contrast, only three individuals had models showing slight attraction to terrestrial agriculture. Terrestrial agriculture, such as cassava and orchards, may be less favored because it lacks suitable microclimate variability to allow for behavioral shifts in the ectothermic Burmese python^58^.

Due to the scope of our research, the underlying drivers of Burmese python’s selection of aquatic features remain unclear; however, one explanation could be the abundance of potential prey items in aquatic environments, such as wading birds which we frequently observed during radio-tracking. Rice paddy fields often act as wetland-like habitat and support diverse wading bird communities^59^. The initial capture of our first tracked individual (PYBI021) occurred after a group of forestry department workers witnessed her consuming several domestic ducks in a small man-made pond. Aquatic environments and the vegetation that typically grows on the edges of ponds and irrigation canals could also serve as suitable refuge in an area highly modified by human settlements, roads, and agricultural practices. Furthermore, irrigation canals could also serve as movement corridors for Burmese pythons as seen with King Cobras that often travel along irrigational canals while in the agricultural land within our study site^30,60^.

Only a single agricultural dwelling individual strongly avoided human settlements. The generally ambivalent movement patterns of most individuals’ merits concern given past records of human-python conflict. Conflict often occurs when wildlife and humans compete for resources, space, and when wildlife causes economic loss through damages or livestock loss^61^—the latter was observed in our study site. Within the agricultural and human-modified areas, many residents keep livestock such as chickens, ducks, and geese. On two occasions, we initially captured study animals after they were seen consuming livestock animals. We captured a third Burmese python after she became entangled in netting material used to enclose a chicken coop. The nature of these captures serves as evidence that Burmese pythons are associated with human-wildlife conflict through preying upon livestock in our study site. Fortunately, none of these instances led to Burmese python persecution, as direct human-snake conflict often results in snake mortality for other species in our study site^62,63^. It is difficult to determine whether Burmese pythons fall victim to persecution in our study site as we suspect that people would hesitate to notify authorities regarding the killing or harm to a protected species. However, we did observe instances of road mortality of non-radio tracked Burmese pythons. Road mortality of Burmese pythons is not entirely surprising as our results suggest that Burmese pythons do not avoid roads, therefore putting individuals at risk of vehicular collision.

While we observed patterns of movement, space use and habitat selection that were fairly uniform across our sample, we acknowledge limited inferences due to several biases our study sample, highlighted by Webster and Rutz^64^. These biases include: acclimation and habituation, trappability, self-selection and genetic make-up.

Acclimation & habituation: We attempted to minimize disturbance to Burmese pythons by limiting tracks to one time per day and by tracking them during periods of inactivity (i.e., during the day). However, our tracking protocol included directly homing in on snakes, it is possible that approaching the snakes could have altered their behaviors and movements over time –either leading to acclimatization to human presence, or driving greater movements to avoid disturbance from researchers.

Trappability and self-selection: Our sampling method depended highly on local residents for notification of Burmese python detections, meaning that our sample likely included animals that more readily entered human settlements. We stress that this sample is non-random and thus possibly not representative of the overall population. We strongly caution extrapolating outside of the sample. We saw considerable differences in both movements and space use between the animals dwelling predominantly in the human dominated matrix compared to the single forest dwelling individual, suggesting that our results may not be generalizable to Burmese pythons in different populations (or even different habitat types).

Genetic make-up: We do not currently have information about the genetic make-up and diversity within our sample size. We tracked several snakes that were captured within close proximity to each other and several could have been related. It is possible that there is little genetic variation in our sample, meaning that our results may not be applicable to more genetically diverse populations of Burmese pythons.

Despite sample limitations we revealed that non-forest pythons showed general selection for aquatic systems and water bodies, which could explain the success of invasive pythons capitalizing on the aquatic environment in the Florida Everglades^65^. The perceived indifference to human dominated spaces suggests we target future work on the interactions between humans and pythons within anthropogenically altered landscapes throughout the region. Specifically, future studies should attempt to quantify instances of human-python conflict and the nature of these conflicts (i.e., persecution, road mortality, livestock loss, property damage). Furthermore, we suggest future studies explore male movements by combining with our publicly accessible data for comparison, further samples of forested individuals could confirm whether lack of site fidelity is the rule and not the exception.

## Conclusion

Our study uses up-to-date, and comprehensive methods to assess Burmese python spatial ecology, providing robust estimates of within study space use, movement patterns and habitat selection in a modified human landscape matrix. Despite our small sample size, we identified several trends in habitat selection and movement. In agricultural land, Burmese pythons limit movement to small areas and appear to remain still for extended time periods. Moves were small, infrequent and associated with water. Limited movement likely influences detection probability of Burmese pythons allowing them to coexist with humans (or at least remain mostly undetected)^66^. Agriculture dwelling Burmese pythons exhibited strong and consistent site fidelity in contrast to invasive Florida Burmese pythons and the single forest-dwelling individual (however we stress that this is a single individual and likely not representative) from our sample.

## Supplementary Information

Supplementary Information.

## Data Availability

The datasets supporting the conclusions of this article are available in the Zenodo repository, http://doi.org/10.5281/zenodo.4026928.
