# Supplementary material for: Native Burmese pythons exhibit site fidelity and preference for aquatic habitats in an agricultural mosaic
Source: Sci Rep. 2021 Mar 29;11:7014. doi: 10.1038/s41598-021-86640-1 (PMC8007826; doi:10.1038/s41598-021-86640-1)
Supplement: Supplementary file 1 — Supplementary Information. [file 41598_2021_86640_MOESM1_ESM.pdf]

Supplementary material for "Native Burmese pythons exhibit site fidelity and preference for aquatic habitats in an agricultural mosaic"

Samantha Nicole Smith[1*], Max Dolton Jones[1], Benjamin Michael Marshall[1], Surachit Waengsothorn[2], George A. Gale[3], Colin Thomas Strine[1**]

[1]School of Biology, Suranaree University of Technology, Nakhon Ratchasima, Thailand

[2]Sakaerat Environmental Research Station, Thailand Institute of Science and Technological Research, Nakhon Ratchasima, Thailand

[3]School of Bioresources and Technology, King Mongkut's University of Technology Thonburi, Bangkok, Thailand

Corresponding author email: *samanthansmith94@gmail.com, ** colin_strine@sut.ac.th

Software/packages used for data analysis

We prepared data for analyses using packages *dplyr v.* 1.0.2 [1], *data.table* v.1.13.0 [2], *reshape2* v.1.4.4 [3], *readr* v.1.3.1 [4], *lubridate* v.1.7.9 [5], and *stringr* v.1.4.0 [6]. We calculated sample means and standard error (mean ± SE) using package *pracma* v.2.2.9 [7]. We used packages *rgdal* v.1.5.16 [8], *raster* v.3.3.13 [9] and *sp* v.1.4.2 [10] to work with rasters and shapefiles. For plot visualization we used *ggplot2* v.3.3.2 [11], *scales* v.1.1.1 [12], *ggthemes* v.4.2.0 [13], *ggspatial* v.1.1.4 [14] and *cowplot* v.1.0.0 [15].

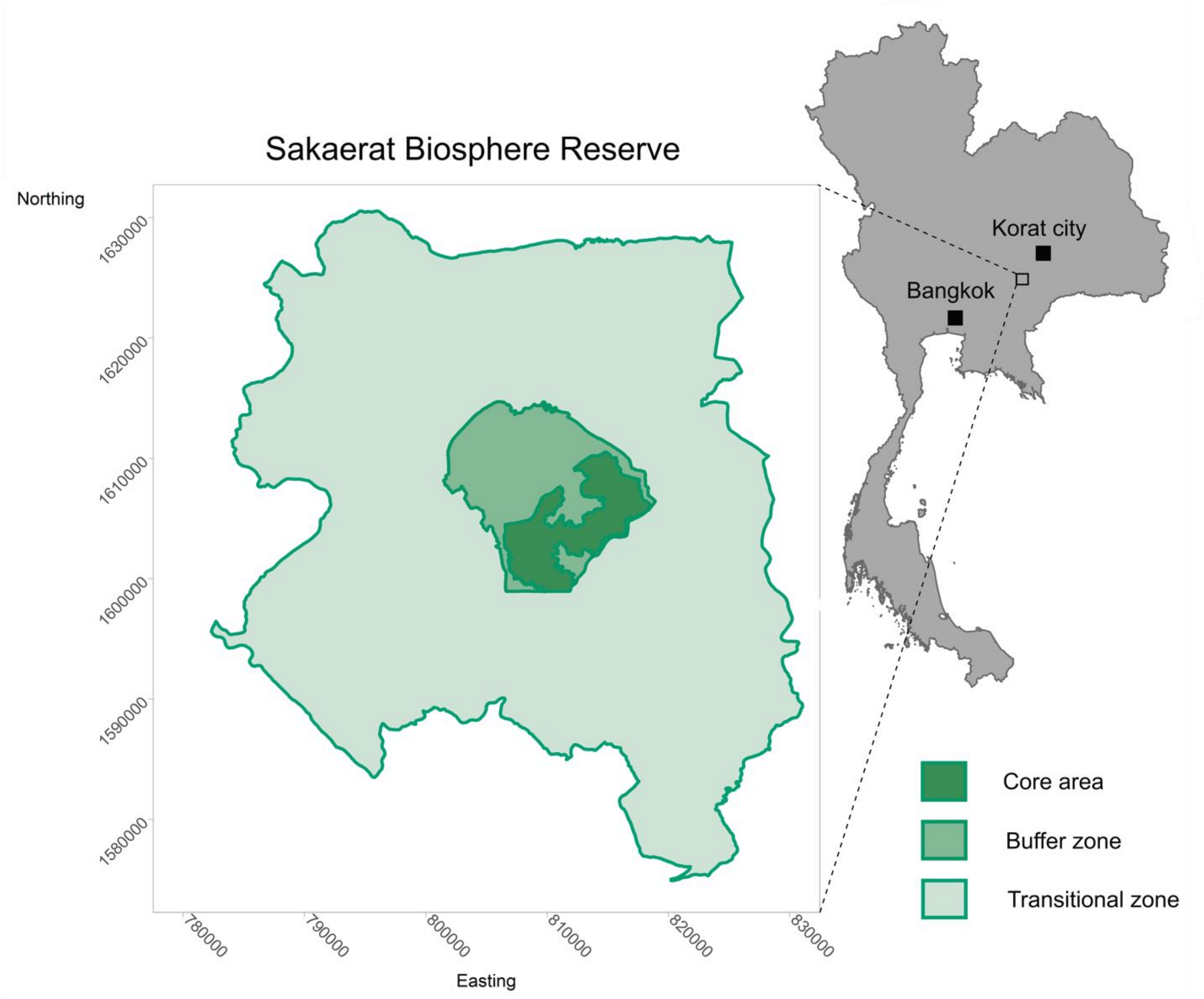

Supplementary Figure 1. A map of the Sakaerat Biosphere Reserve, Nakhon Ratchasima province with an inset map of Thailand highlighting the study site location in relation to Bangkok and Korat city. Map created using R v.3.6.3 (https://www.r-project.org/) in RStudio v.1.2.1335 (https://rstudio.com/) in combination with Inkscape v.1.0.2 (https://inkscape.org/).

Supplementary Table 1. Capture and release dates, coordinates, and methods for all radio-tracked Burmese pythons *(Python bivittatus*) in the Sakaerat Biosphere Reserve, Nakhon Ratchasima, Thailand.

Snake ID	Capture date	Capture (E)	Capture (N)	Capture Method	Release date	Release (E)	Release (N)	Distance (m)
PYBI021	2018-09-24	819617	1607440	Notation	2018-09-28	819808	1607405	193
PYBI022	2018-10-18	820364	1608058	Notation	2018-10-24	820375	1608002	57
PYBI028	2019-01-04	819044	1606437	Opportunistic	2019-01-08	819308	1606727	393
PYBI029	2019-01-31	821671	1609646	Notation	2019-02-22	821671	1609646	0
PYBI033	2019-05-10	819471	1607766	Notation	2019-05-17	819418	1607720	70
PYBI055	2019-11-05	818462	1606647	Notation	2019-11-12	818477	1606766	121
PYBI060	2019-12-26	817051	1605783	Opportunistic	2020-01-05	817241	1605873	210

(E) and (N) refer to UTMs Easting and Northing.

Supplementary Table 2. Biometric measurements of all radio tracked Burmese pythons (*Python bivittatus*) in the Sakaerat Biosphere Reserve, Nakhon Ratchasima, Thailand.

ID	SVL (mm)	TL (mm)	Mass (g)	Sex
PYBI021	2744	330	11240	Female
PYBI022	2304	318	5040	Female
PYBI028	2314	352	7785	Male
PYBI029	2423	300	8660	Female
PYBI033	2214	296	6780	Female
PYBI055	3085	401	19485	Female
PYBI060	2490	352	6965	Female

SVL: Snout to vent length, TL: tail length

Supplementary Table 3. Total dispersal distance and mean dispersal distance per day for all radio-tracked Burmese pythons (*Python bivittatus*) in the Sakaerat Biosphere Reserve, Nakhon Ratchasima, Thailand.

.

Snake ID	Days Tracked	Total dispersal distance (m)	Mean daily displacement (m)
PYBI021	662	20025.42	30.25
PYBI022	486	12864.24	26.47
PYBI028	187	12258.36	65.55
PYBI029	515	27271.92	52.96
PYBI033	41	1455.39	35.50
PYBI055	207	3196.34	15.44
PYBI060	191	12366.29	64.74

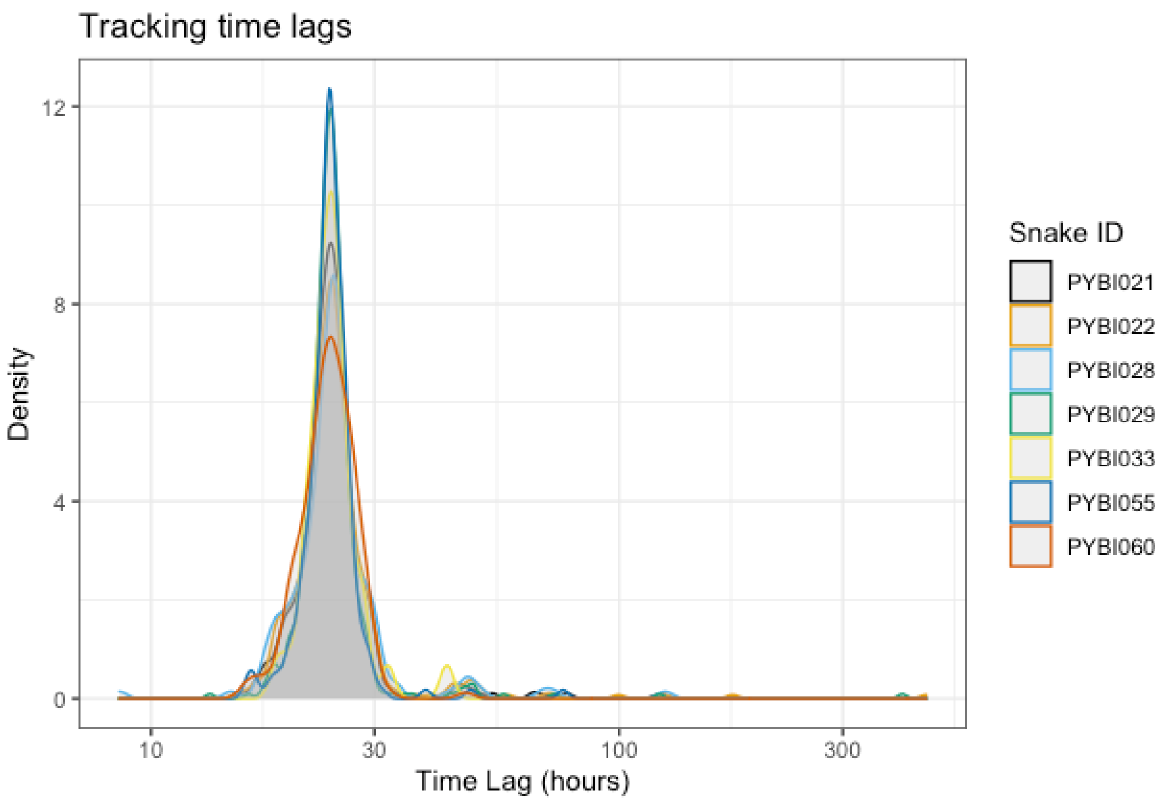

Supplementary Figure 2. Density of time lag between tracks for radio-tracked Burmese pythons *(Python bivittatus*) in the Sakaerat Biosphere Reserve, Nakhon Ratchasima, Thailand.

Supplementary Table 4. Population level ISSF model formulas, mean coefficient estimates (β) and the amount of variation between individual Burmese pythons (*Python bivittatus*) for habitat selection in the Sakaerat Biosphere Reserve, Nakhon Ratchasima, Thailand.

**Model**	**Model formula**	β	**Individual Variance**
1	Step_id + forest + forest:sl + forest:ta	0.000804	0.00000491
2	Step_id + settle + settle:sl + settle:ta	0.000566	0.00000483
3	Step_id + road + road:sl + road:ta	0.001419404	0.00000934
4	Step_id + water + water:sl + water:ta	0.002720069	<0.00000001
5	Step_id + aq.ag + aq.ag:sl + aq.ag:ta	-0.000137	0.0000118
6	Step_id + terr.ag + terr.ag:sl + terr.ag:ta	0.004166886	0.00000817

sl: step length, ta: turn angle, dist_* habitat feature (forest, settle = settlement, road, water, aq. ag= aquatic agriculture, terr.ag = terrestrial agriculture).

Supplementary Table 5. All results from population level ISSF for Burmese pythons (*Python bivittatus*) tracked in the Sakaerat Biosphere Reserve, Nakhon Ratchasima, Thailand.

Mean	Standard Error	Lower Quartile	Median Quartile	Upper Quartile	Mode	Kullback-Leibler Divergence	Term
0.000804	0.000716	-0.000663	0.000811	0.00222805	0.00082	0.000836	dist_forest
0.00000206	0.0000135	-0.000024	0.00000193	0.0000288	0.00000169	0.0000000846	dist_forest:log_sl
-0.000023	0.0000266	-0.0000749	-0.000023	0.0000295	-0.0000232	0.000000583	dist_forest:cos_ta
0.000566	0.00109971	-0.0012233	0.00045	0.00308549	0.000318	0.00340961	dist_settle
0.000000093	0.00000409	-0.00000782	0.0000000577	0.00000821	-0.0000000121	0.00000012	dist_settle:log_sl
-0.00000589	0.00000798	-0.0000215	-0.00000591	0.00000984	-0.00000596	0.000000689	dist_settle:cos_ta
0.0014194	0.00143973	-0.0010807	0.0013038	0.00470286	0.0011849	0.000527	dist_road
0.00000132	0.0000111	-0.0000201	0.00000122	0.0000233	0.00000103	0.000000072	dist_road:log_sl
-0.0000168	0.0000216	-0.000059	-0.0000168	0.0000258	-0.000017	0.000000785	dist_road:cos_ta
0.00272007	0.0006	0.00152945	0.00272433	0.00388592	0.00273284	0.000000354	dist_water
0.000000818	0.00000417	-0.00000727	0.000000783	0.0000091	0.000000713	0.00000021	dist_water:log_sl
-0.00000419	0.00000816	-0.0000201	-0.00000421	0.0000119	-0.00000426	0.000000918	dist_water:cos_ta
-0.000137	0.000773	-0.0016404	-0.000159	0.00148567	-0.000198	0.0000256	dist_aq.ag
-0.0000000587	0.00000197	-0.00000387	-0.0000000755	0.00000385	-0.000000109	0.0000000717	dist_aq.ag:log_sl
-0.00000252	0.00000386	-0.0000101	-0.00000254	0.00000508	-0.00000256	0.000000896	dist_aq.ag:cos_ta
0.00416689	0.00444432	-0.000477	0.00241555	0.01611577	0.00165222	0.0000988	dist_terr.ag
0.000000535	0.00000744	-0.0000139	0.000000471	0.0000153	0.000000345	0.000000331	dist_terr.ag:log_sl
-0.00000922	0.0000146	-0.0000377	-0.00000927	0.0000195	-0.00000937	0.000000532	dist_terr.ag:cos_ta

sl: step length, ta: turn angle, dist_* habitat feature (forest, settle = settlement, road, water, aq. ag= aquatic agriculture, terr.ag = terrestrial agriculture).

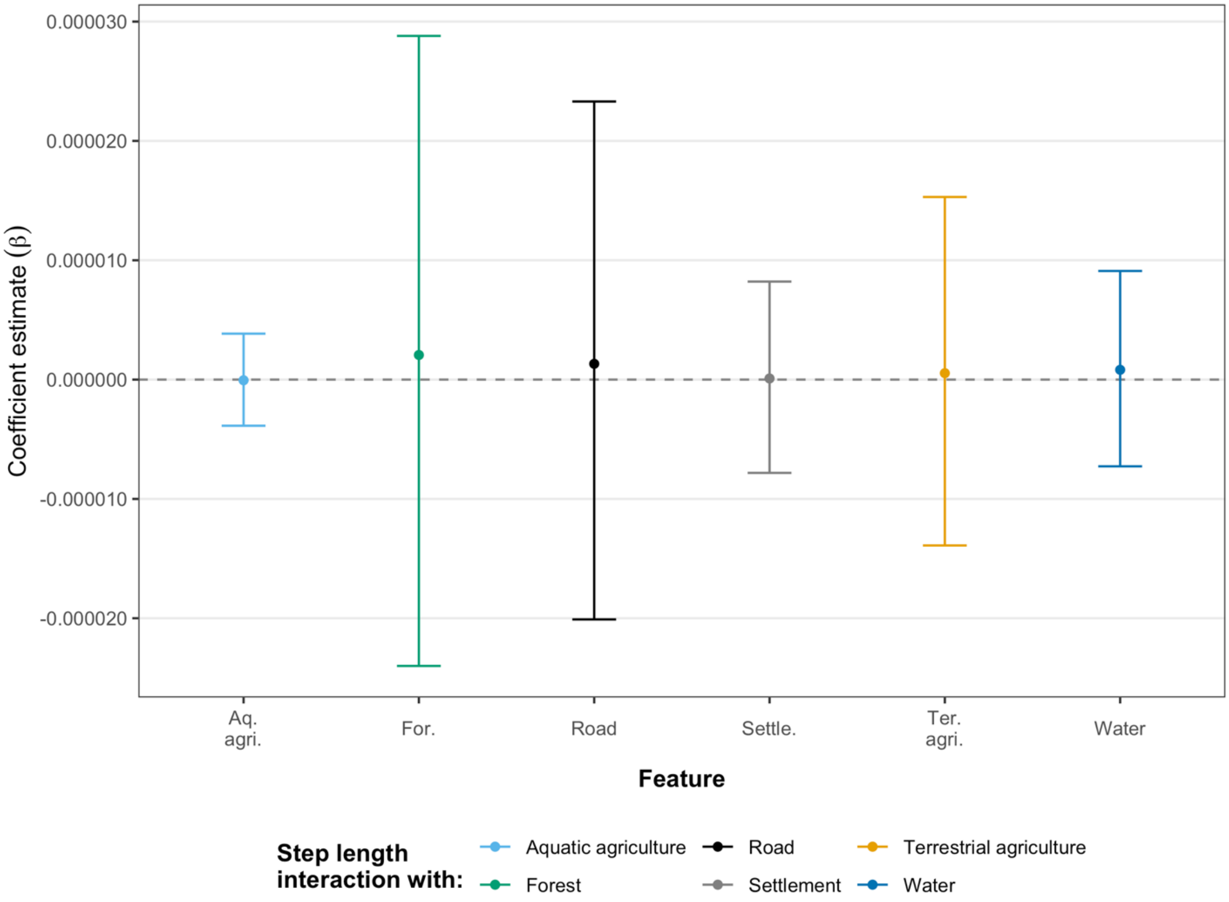

Supplementary figure 3. Interaction between step length and habitat feature at the population level of radio tracked Burmese pythons (*Python bivittatus*) in the Sakaerat Biosphere Reserve, Nakhon Ratchasima, Thailand. Error bars indicate 95% credible intervals.

Supplementary Table 6. All integrated step selection analysis results from individual habitat selection of radio-tracked Burmese pythons (*Python bivittatus*) in the Sakaerat Biosphere Reserve, Nakhon Ratchasima, Thailand.

Term	Estimate	Standard error	Statistic	P value	Confidence Interval (lower)	Confidence Interval (upper)	Snake ID	Model	AIC
log_sl	-0.0202182	0.05279295	-0.3829724	0.70174024	-0.1236905	0.08325405	PYBI021	model1	2238.39539
cos_ta	-0.7006985	0.30182713	-2.3215224	0.02025866	-1.2922688	-0.1091282	PYBI021	model1	2238.39539
log_sl:cos_ta	0.17594274	0.07312053	2.4062017	0.01611936	0.03262914	0.31925634	PYBI021	model1	2238.39539
dist_forest	-0.7797137	2.19599122	-0.3550623	0.72254291	-5.0837774	3.52434999	PYBI021	model2	2235.60231
log_sl	-3.942199	2.31074058	-1.7060327	0.08800199	-8.4711673	0.58676931	PYBI021	model2	2235.60231
cos_ta	11.4486933	4.94707995	2.31423252	0.02065497	1.75259474	21.1447918	PYBI021	model2	2235.60231
dist_forest:log_sl	0.52047042	0.30687779	1.69601854	0.08988237	-0.080999	1.12193984	PYBI021	model2	2235.60231
dist_forest:cos_ta	-1.6190168	0.65812389	-2.4600487	0.01389182	-2.9089159	-0.3291177	PYBI021	model2	2235.60231
log_sl:cos_ta	0.19473642	0.07331735	2.6560754	0.00790559	0.05103705	0.3384358	PYBI021	model2	2235.60231
dist_settle	-3.2158744	9.75831317	-0.3295523	0.74173828	-22.341817	15.9100679	PYBI021	model3	2233.6715
log_sl	-33.483572	13.7288481	-2.4389207	0.0147312	-60.39162	-6.5755243	PYBI021	model3	2233.6715
cos_ta	59.8001653	29.4211492	2.03255709	0.04209729	2.13577262	117.464558	PYBI021	model3	2233.6715
dist_settle:log_sl	3.77920169	1.55081432	2.43691435	0.01481319	0.73966146	6.81874191	PYBI021	model3	2233.6715
dist_settle:cos_ta	-6.8378614	3.32497034	-2.056518	0.03973261	-13.354683	-0.3210393	PYBI021	model3	2233.6715
log_sl:cos_ta	0.19679221	0.07321787	2.68776181	0.00719327	0.05328781	0.3402966	PYBI021	model3	2233.6715
dist_road	-16.122819	9.91713072	-1.6257544	0.10400192	-35.560038	3.31440041	PYBI021	model4	2234.93798
log_sl	-27.046939	15.6996261	-1.722776	0.08492904	-57.817641	3.72376244	PYBI021	model4	2234.93798
cos_ta	84.8967001	31.9839535	2.65435291	0.00794607	22.2093031	147.584097	PYBI021	model4	2234.93798
dist_road:log_sl	3.44970083	2.00415835	1.72127159	0.08520155	-0.4783774	7.37777902	PYBI021	model4	2234.93798
dist_road:cos_ta	-10.933208	4.08510677	-2.676358	0.00744271	-18.93987	-2.9265461	PYBI021	model4	2234.93798
log_sl:cos_ta	0.19551574	0.07381046	2.64888928	0.00807568	0.0508499	0.34018159	PYBI021	model4	2234.93798
dist_water	61.3838945	32.9641944	1.86213847	0.06258357	-3.2247392	125.992528	PYBI021	model5	2221.17615
log_sl	7.72064314	60.7928908	0.12699911	0.8989411	-111.43123	126.87252	PYBI021	model5	2221.17615
cos_ta	61.6967995	114.505698	0.53880986	0.59001806	-162.73024	286.123844	PYBI021	model5	2221.17615
dist_water:log_sl	-0.8738034	6.89134771	-0.1267972	0.89910094	-14.380597	12.63299	PYBI021	model5	2221.17615
dist_water:cos_ta	-7.077632	12.9781907	-0.5453481	0.58551411	-32.514418	18.3591544	PYBI021	model5	2221.17615
log_sl:cos_ta	0.19773297	0.0748163	2.64291309	0.00821961	0.05109572	0.34437021	PYBI021	model5	2221.17615
dist_aq.ag	-7.5683897	22.0253005	-0.3436225	0.73113017	-50.737185	35.600406	PYBI021	model6	2236.23475

log_sl	38.2602767	33.4522051	1.14372959	0.2527358	-27.30484	103.825394	PYBI021	model6	2236.23475
cos_ta	-137.62986	65.0898352	-2.1144601	0.03447599	-265.20359	-10.056129	PYBI021	model6	2236.23475
dist_aq.ag:log_sl	-4.0060551	3.50101838	-1.1442542	0.25251822	-10.867925	2.85581486	PYBI021	model6	2236.23475
dist_aq.ag:cos_ta	14.3286058	6.81148304	2.10359561	0.03541374	0.97834437	27.6788672	PYBI021	model6	2236.23475
log_sl:cos_ta	0.18681099	0.07377316	2.53223517	0.0113338	0.04221825	0.33140373	PYBI021	model6	2236.23475
dist_terr.ag	-20.646123	12.4551158	-1.657642	0.09738974	-45.057702	3.76545511	PYBI021	model7	2237.9397
log_sl	-37.329041	17.4790906	-2.1356398	0.03270879	-71.587429	-3.0706527	PYBI021	model7	2237.9397
cos_ta	82.2276783	46.2088628	1.77947851	0.07516134	-8.3400285	172.795385	PYBI021	model7	2237.9397
dist_terr.ag:log_sl	4.52776133	2.12176804	2.1339568	0.03284632	0.36917239	8.68635028	PYBI021	model7	2237.9397
dist_terr.ag:cos_ta	-10.06951	5.61172019	-1.7943714	0.07275391	-21.06828	0.92925934	PYBI021	model7	2237.9397
log_sl:cos_ta	0.19250606	0.07300832	2.63676872	0.00836999	0.04941238	0.33559974	PYBI021	model7	2237.9397
dist_road	-4.2306909	3.63022512	-1.1654073	0.24385416	-11.345801	2.88441956	PYBI021	model8	2240.91342
dist_forest	0.51781056	1.83208439	0.28263466	0.7774569	-3.0730089	4.10862998	PYBI021	model8	2240.91342
dist_settle	10.6684866	9.33427714	1.14293656	0.25306493	-7.6263604	28.9633336	PYBI021	model8	2240.91342
log_sl	-0.0138324	0.05302224	-0.2608792	0.79418566	-0.1177541	0.09008929	PYBI021	model8	2240.91342
cos_ta	-0.7149013	0.30298637	-2.3595164	0.01829877	-1.3087437	-0.1210589	PYBI021	model8	2240.91342
log_sl:cos_ta	0.18163336	0.0734754	2.47202946	0.01343484	0.03762422	0.3256425	PYBI021	model8	2240.91342
dist_road	-4.1608029	3.49857236	-1.1892859	0.23432717	-11.017879	2.69627292	PYBI021	model9	2219.98671
dist_terr.ag	-2.4344319	7.68392676	-0.3168213	0.75137918	-17.494652	12.6257879	PYBI021	model9	2219.98671
dist_water	56.0128557	12.0371327	4.65333873	0.00000327	32.4205092	79.6052023	PYBI021	model9	2219.98671
log_sl	0.01453099	0.05412152	0.26848815	0.7883236	-0.0915452	0.12060722	PYBI021	model9	2219.98671
cos_ta	-0.7535136	0.3063372	-2.4597522	0.0139033	-1.3539235	-0.1531037	PYBI021	model9	2219.98671
log_sl:cos_ta	0.20187797	0.07459971	2.70614952	0.00680684	0.05566523	0.34809072	PYBI021	model9	2219.98671
dist_water	56.9867599	12.0573055	4.7263263	0.00000229	33.3548755	80.6186444	PYBI021	model10	2216.47897
dist_settle	9.26322559	8.23683455	1.12460989	0.26075438	-6.8806735	25.4071247	PYBI021	model10	2216.47897
dist_aq.ag	-20.242022	15.9346909	-1.2703116	0.20397367	-51.473443	10.9893982	PYBI021	model10	2216.47897
log_sl	0.02402524	0.05464249	0.43968047	0.66016855	-0.0830721	0.13112255	PYBI021	model10	2216.47897
cos_ta	-0.7764358	0.30858792	-2.5160925	0.0118664	-1.381257	-0.1716146	PYBI021	model10	2216.47897
log_sl:cos_ta	0.21009551	0.07528521	2.79066104	0.00526005	0.0625392	0.35765182	PYBI021	model10	2216.47897
log_sl	0.00687405	0.07502795	0.09161982	0.92700011	-0.140178	0.15392613	PYBI022	model1	1564.92228
cos_ta	-0.0561286	0.43750585	-0.1282923	0.89791769	-0.9136243	0.80136711	PYBI022	model1	1564.92228
log_sl:cos_ta	-0.000696	0.10588688	-0.0065712	0.99475698	-0.2082303	0.20683867	PYBI022	model1	1564.92228
dist_forest	1.38935065	3.55881777	0.39039668	0.69624324	-5.585804	8.3645053	PYBI022	model2	1569.43138
log_sl	2.16156858	4.3808922	0.4934083	0.62172412	-6.4248224	10.7479595	PYBI022	model2	1569.43138
cos_ta	7.69971958	7.12657847	1.08042304	0.27995384	-6.2681176	21.6675567	PYBI022	model2	1569.43138
dist_forest:log_sl	-0.2898244	0.5887786	-0.4922467	0.62254492	-1.4438092	0.8641605	PYBI022	model2	1569.43138
dist_forest:cos_ta	-1.0403936	0.95435384	-1.090155	0.27564488	-2.9108928	0.83010555	PYBI022	model2	1569.43138
log_sl:cos_ta	-0.0058205	0.10605791	-0.0548808	0.95623342	-0.2136902	0.20204914	PYBI022	model2	1569.43138
dist_settle	-3.7252969	18.2289486	-0.2043616	0.83807094	-39.45338	32.0027858	PYBI022	model3	1570.53409
log_sl	-8.7616823	28.9445613	-0.3027057	0.76211419	-65.49198	47.9686153	PYBI022	model3	1570.53409

cos_ta	-24.379728	47.9957247	-0.5079562	0.61148403	-118.44962	69.6901639	PYBI022	model3	1570.53409
dist_settle:log_sl	0.98989273	3.26758701	0.30294304	0.76193327	-5.4144601	7.39424559	PYBI022	model3	1570.53409
dist_settle:cos_ta	2.74587604	5.41815885	0.50679135	0.61230123	-7.8735202	13.3652723	PYBI022	model3	1570.53409
log_sl:cos_ta	-0.0011952	0.1060606	-0.0112687	0.99100911	-0.2090701	0.2066798	PYBI022	model3	1570.53409
dist_road	6.65780479	11.9418039	0.55752086	0.57717161	-16.747701	30.0633103	PYBI022	model4	1564.77401
log_sl	-1.3960659	19.3090826	-0.072301	0.94236237	-39.241172	36.4490406	PYBI022	model4	1564.77401
cos_ta	62.595119	32.4253602	1.93043712	0.0535527	-0.9574191	126.147657	PYBI022	model4	1564.77401
dist_road:log_sl	0.18033553	2.46380482	0.07319392	0.94165181	-4.6486332	5.00930425	PYBI022	model4	1564.77401
dist_road:cos_ta	-7.9902295	4.13446361	-1.9325916	0.05328653	-16.093629	0.11317024	PYBI022	model4	1564.77401
log_sl:cos_ta	-0.0081109	0.10748127	-0.0754631	0.93984628	-0.2187703	0.20254856	PYBI022	model4	1564.77401
dist_water	27.1743948	25.9266723	1.04812505	0.29458099	-23.640949	77.9897387	PYBI022	model5	1556.78833
log_sl	-31.591483	47.4978796	-0.6651135	0.50597788	-124.68562	61.5026507	PYBI022	model5	1556.78833
cos_ta	229.373599	93.4314246	2.45499413	0.01408869	46.2513715	412.495826	PYBI022	model5	1556.78833
dist_water:log_sl	3.58497846	5.38599501	0.66561117	0.50565966	-6.9713778	14.1413347	PYBI022	model5	1556.78833
dist_water:cos_ta	-26.016567	10.591908	-2.4562682	0.01403883	-46.776325	-5.2568084	PYBI022	model5	1556.78833
log_sl:cos_ta	0.00998954	0.10894399	0.09169431	0.92694091	-0.2035368	0.22351584	PYBI022	model5	1556.78833
dist_aq.ag	2.55767237	40.5899632	0.06301243	0.9497566	-76.997194	82.1125383	PYBI022	model6	1566.61651
log_sl	-53.143397	67.1060658	-0.7919313	0.42840071	-184.66887	78.3820749	PYBI022	model6	1566.61651
cos_ta	-180.7729	116.350844	-1.5536879	0.12025889	-408.81636	47.2705682	PYBI022	model6	1566.61651
dist_aq.ag:log_sl	5.55439231	7.01260369	0.79205849	0.42832657	-8.1900583	19.298843	PYBI022	model6	1566.61651
dist_aq.ag:cos_ta	18.886416	12.1597147	1.55319565	0.1203764	-4.9461869	42.7190189	PYBI022	model6	1566.61651
log_sl:cos_ta	-0.0085173	0.10669737	-0.0798265	0.93637522	-0.2176403	0.20060572	PYBI022	model6	1566.61651
dist_terr.ag	15.9823863	12.193737	1.31070453	0.18995761	-7.916899	39.8816716	PYBI022	model7	1565.75723
log_sl	15.3763624	18.5726607	0.82790305	0.40772541	-21.025384	51.7781084	PYBI022	model7	1565.75723
cos_ta	55.267858	30.7274383	1.79864841	0.07207431	-4.9568144	115.49253	PYBI022	model7	1565.75723
dist_terr.ag:log_sl	-1.869343	2.2589953	-0.8275108	0.40794761	-6.2968924	2.55820644	PYBI022	model7	1565.75723
dist_terr.ag:cos_ta	-6.7239728	3.73382823	-1.8008254	0.0717304	-14.042142	0.5941961	PYBI022	model7	1565.75723
log_sl:cos_ta	-0.0123328	0.10726163	-0.1149788	0.90846189	-0.2225617	0.19789611	PYBI022	model7	1565.75723
dist_road	6.97147169	4.16174698	1.67513107	0.09390852	-1.1854025	15.1283459	PYBI022	model8	1567.82586
dist_forest	-2.1567274	2.45558063	-0.8782963	0.37978294	-6.969577	2.6561222	PYBI022	model8	1567.82586
dist_settle	5.08816983	11.9496298	0.42580146	0.67025252	-18.332674	28.5090138	PYBI022	model8	1567.82586
log_sl	0.01775669	0.07569174	0.23459218	0.81452528	-0.1305964	0.16610977	PYBI022	model8	1567.82586
cos_ta	-0.0568138	0.4397881	-0.1291844	0.89721174	-0.9187826	0.80515508	PYBI022	model8	1567.82586
log_sl:cos_ta	-0.001069	0.10650322	-0.0100372	0.99199158	-0.2098115	0.20767349	PYBI022	model8	1567.82586
dist_road	2.81290706	4.2663879	0.65931817	0.50969148	-5.5490596	11.1748737	PYBI022	model9	1563.61566
dist_terr.ag	-1.4331337	6.04028788	-0.2372625	0.81245316	-13.27188	10.405613	PYBI022	model9	1563.61566
dist_water	32.4909717	15.2093179	2.13625436	0.03265869	2.68125639	62.300687	PYBI022	model9	1563.61566
log_sl	0.02419953	0.07581295	0.31920052	0.74957446	-0.1243911	0.17279018	PYBI022	model9	1563.61566
cos_ta	-0.0711871	0.44015493	-0.1617319	0.87151701	-0.9338749	0.79150073	PYBI022	model9	1563.61566
log_sl:cos_ta	0.00619522	0.10670131	0.05806134	0.95369977	-0.2029355	0.21532594	PYBI022	model9	1563.61566

dist_water	34.7580359	13.1328867	2.64664096	0.00812956	9.01805092	60.4980209	PYBI022	model10	1562.47217
dist_settle	3.30998176	9.18152602	0.36050453	0.71846987	-14.685479	21.3054421	PYBI022	model10	1562.47217
dist_aq.ag	32.513844	25.8226387	1.25912167	0.20798639	-18.097598	83.1252859	PYBI022	model10	1562.47217
log_sl	0.02792761	0.07601582	0.3673921	0.71332656	-0.1210607	0.17691587	PYBI022	model10	1562.47217
cos_ta	-0.0752928	0.44154144	-0.1705225	0.86459921	-0.9406981	0.79011256	PYBI022	model10	1562.47217
log_sl:cos_ta	0.00757865	0.10707138	0.07078129	0.94357182	-0.2022774	0.21743469	PYBI022	model10	1562.47217
log_sl	-0.0209236	0.08548992	-0.2447492	0.80665062	-0.1884808	0.14663357	PYBI028	model1	725.440246
cos_ta	-0.3120185	0.56937136	-0.5480052	0.58368832	-1.4279658	0.80392888	PYBI028	model1	725.440246
log_sl:cos_ta	0.01718151	0.12126296	0.14168802	0.88732644	-0.2204895	0.25485254	PYBI028	model1	725.440246
dist_forest	-13.14144	6.95386619	-1.8898034	0.05878425	-26.770767	0.48788711	PYBI028	model2	721.248766
log_sl	-23.745182	8.91870276	-2.6624031	0.00775849	-41.225518	-6.2648454	PYBI028	model2	721.248766
cos_ta	18.5859523	18.4651665	1.00654128	0.31415527	-17.605109	54.7770136	PYBI028	model2	721.248766
dist_forest:log_sl	3.05018882	1.14831053	2.65624041	0.00790173	0.79954154	5.3008361	PYBI028	model2	721.248766
dist_forest:cos_ta	-2.4412991	2.38751364	-1.0225278	0.30653116	-7.1207398	2.23814166	PYBI028	model2	721.248766
log_sl:cos_ta	0.04930942	0.11976433	0.41172041	0.68054436	-0.1854243	0.28404319	PYBI028	model2	721.248766
dist_settle	99.9382192	36.1314493	2.76596209	0.00567551	29.1218798	170.754559	PYBI028	model3	720.70499
log_sl	142.534304	50.5672185	2.81870959	0.00482171	43.4243766	241.644231	PYBI028	model3	720.70499
cos_ta	58.5964644	76.5874052	0.7650927	0.44421635	-91.512092	208.70502	PYBI028	model3	720.70499
dist_settle:log_sl	-16.057711	5.69482449	-2.8197025	0.00480682	-27.219362	-4.8960597	PYBI028	model3	720.70499
dist_settle:cos_ta	-6.6230256	8.61103179	-0.7691326	0.44181458	-23.500338	10.2542866	PYBI028	model3	720.70499
log_sl:cos_ta	-0.0113272	0.11981038	-0.0945425	0.92467826	-0.2461512	0.22349687	PYBI028	model3	720.70499
dist_road	34.3933271	17.1614759	2.00410077	0.04505927	0.75745237	68.0292018	PYBI028	model4	726.273322
log_sl	44.0039309	21.2785995	2.06798999	0.03864096	2.29864221	85.7092196	PYBI028	model4	726.273322
cos_ta	-28.002567	40.2754152	-0.6952769	0.48688177	-106.94093	50.9357962	PYBI028	model4	726.273322
dist_road:log_sl	-5.6118473	2.71134717	-2.0697635	0.0384745	-10.92599	-0.2977045	PYBI028	model4	726.273322
dist_road:cos_ta	3.51614179	5.11885381	0.68690022	0.49214561	-6.5166273	13.5489109	PYBI028	model4	726.273322
log_sl:cos_ta	0.04174649	0.12133513	0.34405933	0.73080167	-0.196066	0.27955898	PYBI028	model4	726.273322
dist_water	19.6667045	16.4826121	1.19317887	0.23279933	-12.638622	51.9720306	PYBI028	model5	718.981581
log_sl	48.4360855	20.93623	2.3135056	0.02069486	7.40182865	89.4703423	PYBI028	model5	718.981581
cos_ta	-27.860955	32.3607593	-0.8609487	0.38926628	-91.286877	35.5649681	PYBI028	model5	718.981581
dist_water:log_sl	-5.5240806	2.38664967	-2.3145754	0.02063618	-10.201828	-0.8463332	PYBI028	model5	718.981581
dist_water:cos_ta	3.14025629	3.67697543	0.85403244	0.39308702	-4.0664831	10.3469957	PYBI028	model5	718.981581
log_sl:cos_ta	0.02151365	0.12033992	0.17877398	0.85811518	-0.2143483	0.25737555	PYBI028	model5	718.981581
dist_aq.ag	82.2290043	26.900191	3.0568186	0.002237	29.5055987	134.95241	PYBI028	model6	713.657447
log_sl	133.237688	35.6278308	3.73970811	0.000184	63.4084227	203.066953	PYBI028	model6	713.657447
cos_ta	-29.990319	52.9679952	-0.566197	0.57125988	-133.80568	73.8250438	PYBI028	model6	713.657447
dist_aq.ag:log_sl	-13.966832	3.73270131	-3.7417491	0.000183	-21.282792	-6.6508716	PYBI028	model6	713.657447
dist_aq.ag:cos_ta	3.11036363	5.53606838	0.5618362	0.57422762	-7.740131	13.9608583	PYBI028	model6	713.657447
log_sl:cos_ta	0.01844643	0.11464156	0.1609053	0.87216799	-0.2062469	0.24313976	PYBI028	model6	713.657447
dist_terr.ag	53.1803039	23.7385976	2.24024624	0.02507494	6.65350762	99.7071001	PYBI028	model7	724.979727

log_sl	66.3148964	29.8748174	2.21975905	0.02643513	7.76133014	124.868463	PYBI028	model7	724.979727
cos_ta	-39.483249	49.2259405	-0.8020822	0.42250543	-135.96432	56.9978219	PYBI028	model7	724.979727
dist_terr.ag:log_sl	-8.0481852	3.62363328	-2.2210264	0.02634917	-15.150376	-0.9459944	PYBI028	model7	724.979727
dist_terr.ag:cos_ta	4.74731675	5.96135627	0.79634844	0.42582954	-6.9367268	16.4313603	PYBI028	model7	724.979727
log_sl:cos_ta	0.0374485	0.11928278	0.31394724	0.75356111	-0.1963415	0.27123846	PYBI028	model7	724.979727
dist_road	1.23337189	4.74441909	0.25996268	0.79489256	-8.0655187	10.5322624	PYBI028	model8	728.493989
dist_forest	3.54031349	2.36474395	1.49712339	0.13436115	-1.0944995	8.17512646	PYBI028	model8	728.493989
dist_settle	7.47800017	12.0447532	0.62085126	0.53469749	-16.129282	31.0852826	PYBI028	model8	728.493989
log_sl	-0.0042195	0.08768162	-0.0481227	0.96161845	-0.1760723	0.16763334	PYBI028	model8	728.493989
cos_ta	-0.333216	0.58029812	-0.5742152	0.56582219	-1.4705794	0.80414742	PYBI028	model8	728.493989
log_sl:cos_ta	0.02525474	0.12407921	0.20353725	0.83871514	-0.217936	0.26844553	PYBI028	model8	728.493989
dist_road	2.57895032	5.15864555	0.4999278	0.61712592	-7.5318092	12.6897098	PYBI028	model9	717.278333
dist_terr.ag	26.1027664	9.96278326	2.62002753	0.00879227	6.57607001	45.6294628	PYBI028	model9	717.278333
dist_water	-28.697655	7.94456832	-3.6122359	0.000304	-44.268723	-13.126587	PYBI028	model9	717.278333
log_sl	0.04935492	0.09102987	0.54218383	0.58769187	-0.1290603	0.22777019	PYBI028	model9	717.278333
cos_ta	-0.3536249	0.59241529	-0.5969206	0.55056037	-1.5147375	0.80748772	PYBI028	model9	717.278333
log_sl:cos_ta	0.04357963	0.12726057	0.3424441	0.7320167	-0.2058465	0.29300578	PYBI028	model9	717.278333
dist_water	-18.85846	7.62817639	-2.4722108	0.01342803	-33.809411	-3.9075092	PYBI028	model10	723.75729
dist_settle	13.0586554	11.528857	1.13269298	0.25734321	-9.5374891	35.6547999	PYBI028	model10	723.75729
dist_aq.ag	5.70296338	13.6806578	0.41686324	0.67677844	-21.110633	32.51656	PYBI028	model10	723.75729
log_sl	0.01945757	0.08931862	0.21784451	0.82755026	-0.1556037	0.19451885	PYBI028	model10	723.75729
cos_ta	-0.2812536	0.58492846	-0.4808342	0.63063436	-1.4276923	0.86518512	PYBI028	model10	723.75729
log_sl:cos_ta	0.0119453	0.12496135	0.095592	0.92384462	-0.2329744	0.25686504	PYBI028	model10	723.75729
log_sl	-0.0087511	0.05400022	-0.1620575	0.87126058	-0.1145896	0.09708734	PYBI029	model1	2456.00099
cos_ta	-0.1146397	0.33001649	-0.3473756	0.72830919	-0.7614601	0.53218078	PYBI029	model1	2456.00099
log_sl:cos_ta	0.02653081	0.07566822	0.35062025	0.72587326	-0.1217762	0.1748378	PYBI029	model1	2456.00099
dist_forest	5.20554268	2.32399778	2.23990863	0.02509686	0.65059073	9.76049463	PYBI029	model2	2452.2252
log_sl	2.31726293	2.82021334	0.82166228	0.41126913	-3.2102536	7.8447795	PYBI029	model2	2452.2252
cos_ta	-3.7026587	5.21758597	-0.7096498	0.47792134	-13.928939	6.52362188	PYBI029	model2	2452.2252
dist_forest:log_sl	-0.3014218	0.36691244	-0.8215088	0.41135651	-1.020557	0.41771336	PYBI029	model2	2452.2252
dist_forest:cos_ta	0.46064961	0.67587108	0.68156432	0.49551448	-0.8640334	1.78533259	PYBI029	model2	2452.2252
log_sl:cos_ta	0.04526736	0.07651287	0.59163066	0.55409794	-0.1046951	0.19522983	PYBI029	model2	2452.2252
dist_settle	-10.311896	9.67963631	-1.0653185	0.28673183	-29.283634	8.65984296	PYBI029	model3	2458.8896
log_sl	-7.2078649	14.5255025	-0.4962214	0.61973822	-35.677327	21.2615967	PYBI029	model3	2458.8896
cos_ta	-35.158279	27.7807403	-1.2655631	0.20566955	-89.607529	19.2909712	PYBI029	model3	2458.8896
dist_settle:log_sl	0.82168935	1.65720339	0.49582891	0.62001513	-2.4263696	4.06974831	PYBI029	model3	2458.8896
dist_settle:cos_ta	3.99905445	3.17065275	1.26127166	0.20721099	-2.2153108	10.2134196	PYBI029	model3	2458.8896
log_sl:cos_ta	0.02322476	0.076174	0.30489088	0.76044926	-0.1260735	0.17252305	PYBI029	model3	2458.8896
dist_road	-6.2609726	6.79360583	-0.9215979	0.35673838	-19.576195	7.05425019	PYBI029	model4	2456.14173
log_sl	-17.176373	9.83303622	-1.7468026	0.08067159	-36.44877	2.09602388	PYBI029	model4	2456.14173

cos_ta	7.58900599	19.9394448	0.38060267	0.7034981	-31.491588	46.6695996	PYBI029	model4	2456.14173
dist_road:log_sl	2.19260777	1.25556411	1.74631288	0.0807566	-0.2682527	4.6534682	PYBI029	model4	2456.14173
dist_road:cos_ta	-0.9837735	2.54750561	-0.3861713	0.69936982	-5.9767927	4.00924575	PYBI029	model4	2456.14173
log_sl:cos_ta	0.0279419	0.07618906	0.36674424	0.71380981	-0.1213859	0.1772697	PYBI029	model4	2456.14173
dist_water	7.57884664	23.6030915	0.32109551	0.74813801	-38.682363	53.8400559	PYBI029	model5	2438.10153
log_sl	-62.474843	43.5024033	-1.436124	0.15096707	-147.73799	22.7883004	PYBI029	model5	2438.10153
cos_ta	77.226602	87.6561489	0.88101751	0.37830834	-94.576293	249.029497	PYBI029	model5	2438.10153
dist_water:log_sl	7.08919725	4.93474983	1.43658696	0.15083539	-2.5827347	16.7611292	PYBI029	model5	2438.10153
dist_water:cos_ta	-8.7740604	9.94147124	-0.8825716	0.37746776	-28.258986	10.7108651	PYBI029	model5	2438.10153
log_sl:cos_ta	0.0344535	0.0773436	0.44546021	0.65598721	-0.1171372	0.18604416	PYBI029	model5	2438.10153
dist_aq.ag	73.9615925	127.398309	0.58055396	0.56154111	-175.73451	323.65769	PYBI029	model6	2460.39387
log_sl	70.9554158	240.851425	0.29460243	0.7682976	-401.1047	543.015535	PYBI029	model6	2460.39387
cos_ta	-149.09173	441.485897	-0.3377044	0.73558596	-Inf	Inf	PYBI029	model6	2460.39387
dist_aq.ag:log_sl	-7.411162	25.1541833	-0.2946294	0.768277	-56.712455	41.8901314	PYBI029	model6	2460.39387
dist_aq.ag:cos_ta	15.5585616	46.1069415	0.3374451	0.73578139	-74.809383	105.926506	PYBI029	model6	2460.39387
log_sl:cos_ta	0.026992	0.07584855	0.35586706	0.72194012	-0.1216684	0.17565242	PYBI029	model6	2460.39387
dist_terr.ag	-12.937223	10.0539277	-1.286783	0.19816993	-32.64256	6.76811285	PYBI029	model7	2453.68405
log_sl	-33.903992	15.4207964	-2.1985889	0.02790717	-64.128197	-3.6797861	PYBI029	model7	2453.68405
cos_ta	-0.9600174	30.7558044	-0.0312142	0.97509873	-61.240286	59.3202516	PYBI029	model7	2453.68405
dist_terr.ag:log_sl	4.12101839	1.87491621	2.19797469	0.02795091	0.44625013	7.79578664	PYBI029	model7	2453.68405
dist_terr.ag:cos_ta	0.10240193	3.74144751	0.0273696	0.97816494	-7.2307004	7.4355043	PYBI029	model7	2453.68405
log_sl:cos_ta	0.02843651	0.07614626	0.37344588	0.70881662	-0.1208074	0.17768043	PYBI029	model7	2453.68405
dist_road	3.0934998	2.79986791	1.10487348	0.26921442	-2.3941405	8.58114007	PYBI029	model8	2444.86425
dist_forest	5.60014727	1.59959253	3.50098363	4.64E-04	2.46500352	8.73529102	PYBI029	model8	2444.86425
dist_settle	-13.620161	5.36282648	-2.5397355	0.01109363	-24.131108	-3.1092142	PYBI029	model8	2444.86425
log_sl	0.02206751	0.05566456	0.39643734	0.69178243	-0.087033	0.13116803	PYBI029	model8	2444.86425
cos_ta	-0.1870379	0.33929673	-0.5512518	0.58146108	-0.8520473	0.47797144	PYBI029	model8	2444.86425
log_sl:cos_ta	0.05185503	0.07831183	0.66216091	0.5078681	-0.1016333	0.2053434	PYBI029	model8	2444.86425
dist_road	2.88400082	2.98867699	0.96497575	0.33455696	-2.9736984	8.74170008	PYBI029	model9	2439.77213
dist_terr.ag	-1.8116594	4.96323709	-0.3650157	0.7150997	-11.539425	7.91610658	PYBI029	model9	2439.77213
dist_water	36.9302665	8.8026761	4.19534538	0.0000272	19.6773384	54.1831946	PYBI029	model9	2439.77213
log_sl	0.02671521	0.0557138	0.47950798	0.63157729	-0.0824818	0.13591226	PYBI029	model9	2439.77213
cos_ta	-0.1387167	0.33670905	-0.4119779	0.68035561	-0.7986543	0.52122092	PYBI029	model9	2439.77213
log_sl:cos_ta	0.03650899	0.07737083	0.47187017	0.63701946	-0.1151351	0.18815303	PYBI029	model9	2439.77213
dist_water	37.3677028	8.26678011	4.5202246	0.00000618	21.1651115	53.5702941	PYBI029	model10	2437.96269
dist_settle	-6.6292859	4.5756286	-1.4488252	0.14738641	-15.597353	2.33878135	PYBI029	model10	2437.96269
dist_aq.ag	30.7522055	37.8806893	0.81181747	0.41689637	-43.492581	104.996992	PYBI029	model10	2437.96269
log_sl	0.02993998	0.05584071	0.53616753	0.59184277	-0.0795058	0.13938576	PYBI029	model10	2437.96269
cos_ta	-0.1341825	0.33717225	-0.3979644	0.69065644	-0.795028	0.52666292	PYBI029	model10	2437.96269
log_sl:cos_ta	0.0345758	0.07744502	0.44645606	0.65526785	-0.1172136	0.18636524	PYBI029	model10	2437.96269

log_sl	-0.0229963	0.21801209	-0.1054816	0.91599365	-0.4502921	0.40429958	PYBI033	model1	228.63292
cos_ta	-0.3388679	1.21385158	-0.2791675	0.78011629	-2.7179733	2.04023748	PYBI033	model1	228.63292
log_sl:cos_ta	0.09685293	0.30980569	0.31262475	0.75456577	-0.5103551	0.70406093	PYBI033	model1	228.63292
dist_forest	-4.2820497	16.3488177	-0.261918	0.79338466	-36.325144	27.7610442	PYBI033	model2	232.58281
log_sl	-12.016826	21.8179407	-0.5507773	0.58178636	-54.779204	30.7455521	PYBI033	model2	232.58281
cos_ta	44.3215748	33.3249605	1.32998132	0.18352443	-20.994148	109.637297	PYBI033	model2	232.58281
dist_forest:log_sl	1.55339527	2.82869664	0.54915584	0.58289851	-3.9907483	7.09753881	PYBI033	model2	232.58281
dist_forest:cos_ta	-5.8068517	4.32791818	-1.3417194	0.179687	-14.289415	2.67571202	PYBI033	model2	232.58281
log_sl:cos_ta	0.14408659	0.3218666	0.44765933	0.65439908	-0.4867604	0.77493353	PYBI033	model2	232.58281
dist_settle	-117.26178	103.157716	-1.1367233	0.25565399	-319.44718	84.9236334	PYBI033	model3	228.086243
log_sl	-357.97581	204.568062	-1.7499106	0.08013375	-Inf	42.9702216	PYBI033	model3	228.086243
cos_ta	-555.76324	380.028932	-1.4624235	0.14362519	-Inf	189.079778	PYBI033	model3	228.086243
dist_settle:log_sl	40.3236372	23.0481256	1.74954084	0.08019758	-4.849859	85.4971334	PYBI033	model3	228.086243
dist_settle:cos_ta	62.6198308	42.8531023	1.46126715	0.14394214	-21.370706	146.610368	PYBI033	model3	228.086243
log_sl:cos_ta	-0.0522855	0.32354021	-0.1616043	0.87161744	-0.6864127	0.58184166	PYBI033	model3	228.086243
dist_road	-20.472192	36.3084358	-0.5638412	0.57286222	-91.635418	50.6910349	PYBI033	model4	227.849348
log_sl	-74.731384	66.7851553	-1.118982	0.26314783	-205.62788	56.165115	PYBI033	model4	227.849348
cos_ta	-194.64148	105.339135	-1.8477603	0.06463702	-401.10239	11.8194349	PYBI033	model4	227.849348
dist_road:log_sl	9.52675324	8.51808475	1.11841494	0.26338982	-7.1683861	26.2218926	PYBI033	model4	227.849348
dist_road:cos_ta	24.8168885	13.4555193	1.84436498	0.06512997	-1.5554446	51.1892217	PYBI033	model4	227.849348
log_sl:cos_ta	-0.0149157	0.33428322	-0.0446198	0.96441034	-0.6700987	0.64026741	PYBI033	model4	227.849348
dist_water	31.8768386	171.78583	0.18556151	0.85278864	-304.8172	368.570879	PYBI033	model5	228.754028
log_sl	-22.566726	340.175508	-0.0663385	0.94710835	-689.29847	644.165019	PYBI033	model5	228.754028
cos_ta	-976.07864	480.035967	-2.0333448	0.0420177	-Inf	-35.225428	PYBI033	model5	228.754028
dist_water:log_sl	2.56055821	38.562426	0.06640034	0.9470591	-73.020408	78.1415243	PYBI033	model5	228.754028
dist_water:cos_ta	110.586939	54.4085405	2.03252905	0.04210013	3.94815948	217.225719	PYBI033	model5	228.754028
log_sl:cos_ta	0.16228182	0.33641208	0.48238998	0.62952894	-0.4970737	0.82163737	PYBI033	model5	228.754028
dist_aq.ag	10.3301693	123.044056	0.08395504	0.93309217	-230.83175	251.492088	PYBI033	model6	227.177794
log_sl	334.281679	219.151886	1.52534246	0.1271737	-95.248126	Inf	PYBI033	model6	227.177794
cos_ta	-547.55062	373.966334	-1.4641709	0.14314726	-Inf	185.409925	PYBI033	model6	227.177794
dist_aq.ag:log_sl	-35.056521	22.9786377	-1.5256135	0.12710614	-80.093823	9.98078141	PYBI033	model6	227.177794
dist_aq.ag:cos_ta	57.311756	39.1919598	1.46233453	0.14364956	-19.503074	134.126586	PYBI033	model6	227.177794
log_sl:cos_ta	0.36848326	0.32227066	1.14339685	0.25287386	-0.2631556	1.00012215	PYBI033	model6	227.177794
dist_terr.ag	-70.26502	188.214191	-0.3733248	0.70890674	-439.15806	298.628016	PYBI033	model7	230.81822
log_sl	-321.60062	366.018224	-0.8786465	0.379593	-Inf	395.781919	PYBI033	model7	230.81822
cos_ta	768.731982	611.790979	1.25652716	0.2089249	-430.3563	Inf	PYBI033	model7	230.81822
dist_terr.ag:log_sl	38.9821701	44.3726063	0.87851883	0.37966222	-47.98654	125.95088	PYBI033	model7	230.81822
dist_terr.ag:cos_ta	-93.261975	74.1880809	-1.2571019	0.20871675	-238.66794	52.1439913	PYBI033	model7	230.81822
log_sl:cos_ta	0.18729939	0.31518815	0.5942463	0.55234739	-0.430458	0.80505681	PYBI033	model7	230.81822
dist_road	15.629678	19.7877856	0.78986494	0.42960665	-23.153669	54.4130252	PYBI033	model8	232.650963

dist_forest	3.33018331	9.06599582	0.36732681	0.71337526	-14.438842	21.0992086	PYBI033	model8	232.650963
dist_settle	15.095733	61.5880184	0.24510828	0.80637259	-105.61456	135.806031	PYBI033	model8	232.650963
log_sl	-0.0235624	0.21965933	-0.1072679	0.91457644	-0.4540868	0.40696198	PYBI033	model8	232.650963
cos_ta	-0.3816629	1.20931255	-0.3156032	0.75230371	-2.7518719	1.98854614	PYBI033	model8	232.650963
log_sl:cos_ta	0.11668068	0.31132203	0.37479095	0.70781595	-0.4934993	0.72686064	PYBI033	model8	232.650963
dist_road	27.8685301	14.2284833	1.95864377	0.05015452	-0.0187847	55.7558449	PYBI033	model9	227.458143
dist_terr.ag	101.67941	56.24233	1.80788047	0.07062511	-8.553531	211.912351	PYBI033	model9	227.458143
dist_water	61.2903038	46.326995	1.3229933	0.18583761	-29.508938	152.089545	PYBI033	model9	227.458143
log_sl	0.02960912	0.22714847	0.13035138	0.89628843	-0.4155937	0.47481194	PYBI033	model9	227.458143
cos_ta	-0.2463053	1.23181263	-0.1999536	0.8415169	-2.6606137	2.16800307	PYBI033	model9	227.458143
log_sl:cos_ta	0.11040699	0.31564546	0.34978165	0.72650257	-0.5082467	0.72906072	PYBI033	model9	227.458143
dist_water	105.485013	54.7558557	1.92646086	0.05404686	-1.8344925	212.804518	PYBI033	model10	226.236962
dist_settle	48.9340782	46.3008987	1.05687102	0.29057046	-41.814016	139.682172	PYBI033	model10	226.236962
dist_aq.ag	-155.44382	73.9984563	-2.100636	0.03567293	-300.47813	-10.40951	PYBI033	model10	226.236962
log_sl	0.06900916	0.23030808	0.29963845	0.76445295	-0.3823864	0.5204047	PYBI033	model10	226.236962
cos_ta	-0.8481072	1.26030058	-0.6729404	0.50098522	-3.3182509	1.62203658	PYBI033	model10	226.236962
log_sl:cos_ta	0.32672205	0.33414175	0.97779475	0.32817585	-0.3281837	0.98162784	PYBI033	model10	226.236962
log_sl	-0.0407311	0.12555807	-0.3244005	0.74563487	-0.2868204	0.2053582	PYBI055	model1	269.387671
cos_ta	-0.9878936	0.74426569	-1.32734	0.18439624	-2.4466276	0.47084034	PYBI055	model1	269.387671
log_sl:cos_ta	0.22433114	0.17344246	1.29340378	0.19587144	-0.1156098	0.56427212	PYBI055	model1	269.387671
dist_forest	11.4855746	12.4871034	0.91979495	0.35767992	-12.988698	35.9598474	PYBI055	model2	273.901152
log_sl	9.40481603	17.7227827	0.53066249	0.59565268	-25.3312	44.1408319	PYBI055	model2	273.901152
cos_ta	11.6693923	36.596361	0.31886756	0.74982695	-60.058157	83.3969419	PYBI055	model2	273.901152
dist_forest:log_sl	-1.2101226	2.26909132	-0.5333071	0.59382099	-5.6574599	3.23721468	PYBI055	model2	273.901152
dist_forest:cos_ta	-1.6163304	4.68075409	-0.3453141	0.72985824	-10.79044	7.55777902	PYBI055	model2	273.901152
log_sl:cos_ta	0.21609188	0.173191	1.24770853	0.21213781	-0.1233562	0.55554	PYBI055	model2	273.901152
dist_settle	68.8951997	51.3658166	1.34126554	0.17983425	-31.779951	169.57035	PYBI055	model3	269.769705
log_sl	73.7023551	83.1878529	0.88597497	0.37563102	-89.342841	236.747551	PYBI055	model3	269.769705
cos_ta	334.341424	191.659763	1.74445287	0.08108015	-41.30481	Inf	PYBI055	model3	269.769705
dist_settle:log_sl	-8.3079573	9.37237905	-0.8864299	0.3753859	-26.677483	10.0615681	PYBI055	model3	269.769705
dist_settle:cos_ta	-37.750167	21.5796709	-1.7493393	0.08023238	-80.045545	4.54521075	PYBI055	model3	269.769705
log_sl:cos_ta	0.12824743	0.18528115	0.69217743	0.48882591	-0.234897	0.49139182	PYBI055	model3	269.769705
dist_road	66.1428375	35.0596467	1.88658026	0.0592168	-2.5728074	134.858482	PYBI055	model4	267.634621
log_sl	66.2305901	50.3810234	1.31459398	0.18864639	-32.514401	164.975582	PYBI055	model4	267.634621
cos_ta	192.555114	119.755888	1.60789684	0.10785777	-42.162114	427.272341	PYBI055	model4	267.634621
dist_road:log_sl	-8.4362662	6.41095667	-1.3159138	0.18820298	-21.00151	4.12897795	PYBI055	model4	267.634621
dist_road:cos_ta	-24.602038	15.2254994	-1.6158444	0.10612796	-54.443468	5.23939251	PYBI055	model4	267.634621
log_sl:cos_ta	0.13948193	0.18054502	0.77256037	0.4397826	-0.2143798	0.49334367	PYBI055	model4	267.634621
dist_water	196.408748	133.314021	1.47327901	0.14067582	-64.881931	457.699427	PYBI055	model5	258.010249
log_sl	110.670247	232.186039	0.47664471	0.63361514	-344.40603	565.746521	PYBI055	model5	258.010249

cos_ta	485.696224	482.450009	1.0067286	0.31406521	-459.88842	Inf	PYBI055	model5	258.010249
dist_water:log_sl	-12.525599	26.3121062	-0.4760394	0.63404631	-64.09638	39.0451811	PYBI055	model5	258.010249
dist_water:cos_ta	-55.142527	54.6582371	-1.0088603	0.31304163	-162.2707	51.9856496	PYBI055	model5	258.010249
log_sl:cos_ta	0.24183342	0.20061972	1.20543196	0.22803659	-0.151374	0.63504085	PYBI055	model5	258.010249
dist_aq.ag	130.650766	72.0800944	1.81257762	0.06989699	-10.623623	271.925155	PYBI055	model6	268.804548
log_sl	103.867681	117.898287	0.88099398	0.37832108	-127.20871	334.944077	PYBI055	model6	268.804548
cos_ta	369.594919	274.35733	1.34712974	0.17793845	-168.13557	Inf	PYBI055	model6	268.804548
dist_aq.ag:log_sl	-10.860402	12.3234986	-0.8812759	0.37816853	-35.014015	13.2932116	PYBI055	model6	268.804548
dist_aq.ag:cos_ta	-38.720434	28.6643872	-1.3508202	0.17675302	-94.901601	17.4607323	PYBI055	model6	268.804548
log_sl:cos_ta	0.21461585	0.18864062	1.13769694	0.25524705	-0.155113	0.58434467	PYBI055	model6	268.804548
dist_terr.ag	28.6344599	84.9556207	0.33705198	0.73607772	-137.8755	195.144417	PYBI055	model7	272.421182
log_sl	-40.964677	146.062755	-0.2804594	0.77912505	-327.24242	245.313062	PYBI055	model7	272.421182
cos_ta	163.559284	337.12617	0.48515748	0.62756464	-497.19587	Inf	PYBI055	model7	272.421182
dist_terr.ag:log_sl	4.96288186	17.7098622	0.28023266	0.77929901	-29.74781	39.6735739	PYBI055	model7	272.421182
dist_terr.ag:cos_ta	-19.956074	40.8790872	-0.4881732	0.62542718	-100.07761	60.1654648	PYBI055	model7	272.421182
log_sl:cos_ta	0.24816496	0.17528019	1.4158186	0.15682862	-0.0953779	0.59170783	PYBI055	model7	272.421182
dist_road	14.4419514	12.3852079	1.16606451	0.24358837	-9.8326101	38.7165129	PYBI055	model8	271.584402
dist_forest	7.25917516	5.32418806	1.36343327	0.172746	-3.1760417	17.694392	PYBI055	model8	271.584402
dist_settle	12.454259	21.6497736	0.57526047	0.56511515	-29.978518	54.8870356	PYBI055	model8	271.584402
log_sl	-0.0329513	0.12812946	-0.2571716	0.79704633	-0.2840804	0.21817788	PYBI055	model8	271.584402
cos_ta	-0.9635034	0.74982472	-1.2849715	0.19880224	-2.4331328	0.50612604	PYBI055	model8	271.584402
log_sl:cos_ta	0.21636178	0.17592373	1.22986125	0.21874907	-0.1284424	0.56116595	PYBI055	model8	271.584402
dist_road	23.2067544	11.7387785	1.97693094	0.04804944	0.19917138	46.2143374	PYBI055	model9	252.337735
dist_terr.ag	58.7199749	33.0911006	1.77449447	0.0759814	-6.1373904	123.57734	PYBI055	model9	252.337735
dist_water	117.059666	33.2396342	3.52168935	4.29E-04	51.9111798	182.208152	PYBI055	model9	252.337735
log_sl	0.18807245	0.15619405	1.20409482	0.22855293	-0.1180623	0.49420717	PYBI055	model9	252.337735
cos_ta	-1.2282692	0.8069363	-1.522139	0.12797425	-2.8098353	0.35329686	PYBI055	model9	252.337735
log_sl:cos_ta	0.34638243	0.19155647	1.80825231	0.07056724	-0.0290614	0.7218262	PYBI055	model9	252.337735
dist_water	108.32889	34.2818676	3.15994715	0.00157798	41.1376638	175.520115	PYBI055	model10	258.7288
dist_settle	15.5305463	23.863534	0.65080663	0.51517132	-31.241121	62.3022135	PYBI055	model10	258.7288
dist_aq.ag	11.0232775	31.2555182	0.3526826	0.7243264	-50.236413	72.2829676	PYBI055	model10	258.7288
log_sl	0.14660393	0.15137333	0.96849242	0.3327985	-0.1500824	0.44329021	PYBI055	model10	258.7288
cos_ta	-1.1462094	0.81309141	-1.4096931	0.15863031	-2.7398393	0.44742051	PYBI055	model10	258.7288
log_sl:cos_ta	0.31746086	0.1951068	1.62711327	0.10371304	-0.0649414	0.69986315	PYBI055	model10	258.7288

sl: step length, ta: turn angle, dist_* habitat feature (forest, settle = settlement, road, water, aq. ag= aquatic agriculture, terr.ag = terrestrial agriculture).

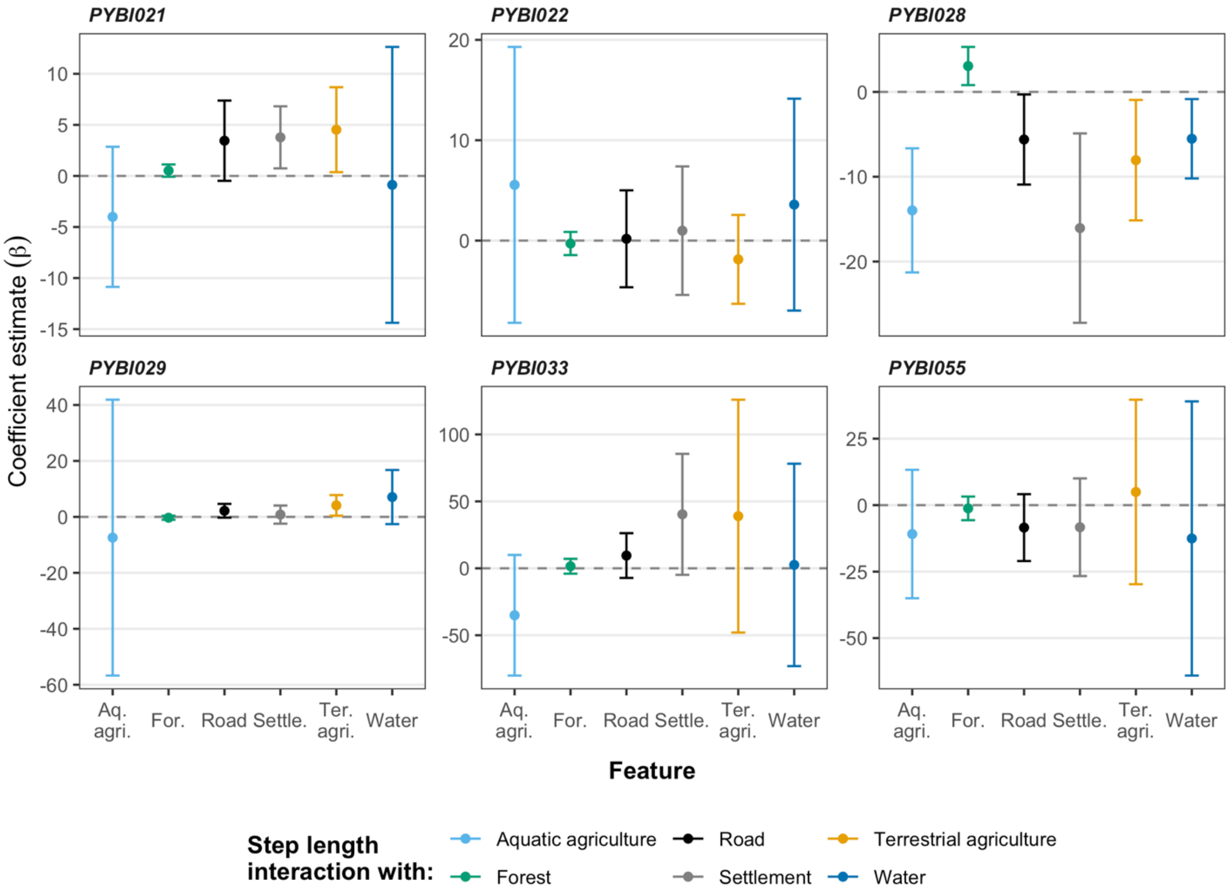

Supplementary Figure 4. Interaction between step length and habitat feature at the individual level for radio tracked Burmese pythons (*Python bivittatus*) in the Sakaerat Biosphere Reserve, Nakhon Ratchasima, Thailand. Error bars indicate 95% credible intervals.

References

1. Wickham, H., François, R., Henry, L. & Müller, K. *dplyr: A Grammar of Data Manipulation*. (2020).
2. Dowle, M. & Srinivasan, A. *data.table: Extension of `data.frame`*. (2020).
3. Wickham, H. Reshaping Data with the reshape Package. *J. Stat. Softw.* **21**, 1–20 (2007).
4. Wickham, H., Hester, J. & Francois, R. *readr: Read Rectangular Text Data*. (2018).

5. Grolemund, G. & Wickham, H. Dates and Times Made Easy with lubridate. *J. Stat. Softw.* **40**, 1–25 (2011).
6. Wickham, H. *stringr: Simple, Consistent Wrappers for Common String Operations*. (2019).
7. Borchers, H. W. *pracma: Practical Numerical Math Functions*. (2019).
8. Bivand, R., Keitt, T. & Rowlingson, B. *rgdal: Bindings for the 'Geospatial' Data Abstraction Library*. (2020).
9. Hijmans, R. J. *raster: Geographic Data Analysis and Modeling*. (2020).
10. Bivand, R. S., Pebesma, E. & Gomez-Rubio, V. *Applied spatial data analysis with R, Second edition*. (Springer, NY, 2013).
11. Wickham, H. *ggplot2: Elegant Graphics for Data Analysis*. (Springer-Verlag New York, 2016).
12. Wickham, H. & Seidel, D. *scales: Scale Functions for Visualization*. (2020).
13. Arnold, J. B. *ggthemes: Extra Themes, Scales and Geoms for 'ggplot2'*. (2019).
14. Dunnington, D. *ggspatial: Spatial Data Framework for ggplot2*. (2020).
15. Wilke, C. O. *cowplot: Streamlined Plot Theme and Plot Annotations for 'ggplot2'*. (2019).
